# Exploring the link between women’s empowerment, depression, and anxiety in Bangladesh: evidence from a nationally representative cross-sectional study

**DOI:** 10.7189/jogh.16.04189

**Published:** 2026-05-29

**Authors:** Yun-Jung Eom, Sion Jang, Andreas Hasman, S V Subramanian, Rockli Kim

**Affiliations:** 1 Interdisciplinary Program in Precision Public Health, Department of Public Health Sciences, Graduate School of Korea University, Seoul, South Korea; 2 UNICEF Programme Division, New York, New York, USA; 3 Harvard Centre for Population and Development Studies, Cambridge, Massachusetts, USA; 4 Department of Social and Behavioural Sciences, Harvard T. H. Chan School of Public Health, Boston, Massachusetts, USA; 5 Division of Health Policy and Management, College of Health Science, Korea University, Seoul, South Korea

## Abstract

**Background:**

Women’s mental health remains critically under-researched in Bangladesh, despite its close links with gender inequality. In this study, we examined how three domains of women’s empowerment – attitudes to violence, social independence, and decision-making – are associated with mental health outcomes among currently married women in Bangladesh.

**Methods:**

Using nationally representative data from the 2022 Bangladesh Demographic and Health Survey, we analysed 18 900 currently married women. We assessed women’s empowerment using the survey-based women’s empowerment index, which was categorised as low, medium, or high. Depression and anxiety were measured using the Patient Health Questionnaire-9 and the Generalised Anxiety Disorder-7, respectively. We applied multivariable logistic regression models, adjusting for key sociodemographic covariates.

**Results:**

Overall, 4.6% women reported depression, and 4.0% reported anxiety. Women with low empowerment in attitude to violence domain had higher odds of depression (adjusted odds ratio (aOR) = 1.48; 95% confidence interval (CI) = 1.17–1.86) and anxiety (aOR = 1.41; 95% CI = 1.09–1.82), compared to women with high empowerment. In the social independence domain, low empowerment was significantly associated with anxiety (aOR = 1.28; 95% CI = 1.02–1.60) but not with depression. Low empowerment in the decision-making domain was not associated with depressive or anxiety symptoms; however, it was associated with lower odds of severe depressive (aOR = 0.48; 95% CI = 0.33–0.70) and anxiety symptoms (aOR = 0.44; 95% CI = 0.28–0.71) when the outcome was disaggregated by severity.

**Conclusions:**

Women’s empowerment is associated with mental health in Bangladesh. Structural interventions must integrate gender-transformative approaches that challenge discriminatory norms and promote women’s social standing, reinforcing the interconnection between Sustainable Development Goal three (good health and well-being) and five (gender equality).

Mental health is increasingly recognised as an essential component of overall health and well-being, carrying profound implications for social and economic development [1]. The Lancet Commission on Global Mental Health and Sustainable Development underscores mental health as a fundamental human right that demands urgent attention, particularly in low- and middle- income countries (LMICs) where the right to care and protection is often severely undermined [2]. Both globally as well as in the context of LMICs, women are disproportionately affected by mental disorders, exhibiting a consistently higher prevalence of mental distress compared to men [3–5]. The Global Burden of Disease Study 2019, for instance, estimated the global prevalence of depressive disorders at 4.2% among women and 2.7% among men, and anxiety disorders at 4.7% among women and 2.9% among men [4].

While biological and reproductive factors partially contribute to this disparity, a substantial body of literature underscores the role of systemic gender inequalities characterised by women’s low social status, gender-based violence, and rigid gender norms in shaping women’s mental health outcomes [6–8]. These structural and normative constraints not only elevate women’s vulnerability to common mental disorders such as depression and anxiety but also restrict their access to mental health resources [9], perpetuating a cycle of disadvantages. In this context, women’s (dis)empowerment – both as a manifestation of gendered power imbalances and as a potential lever for achieving greater gender equity – emerges as a critical determinant of women’s mental health. Notably, recent studies in the context of LMICs have shown that women’s greater empowerment is negatively associated with their mental distress [10–15]. A longitudinal study in rural Burkina Faso, for example, found that women who refused to justify their husbands’ wife-beating were significantly less likely to experience high stress [11]. Similarly, a study in Iran reported that improvements in women’s health literacy reduced psychological distress and social dysfunction [16]. Similarly, a cross-sectional study in rural India identified a protective role of women’s decision-making power against common mental disorders [12].

However, existing evidence in LMICs faces several methodological limitations. First, many studies relied on subnational data, limiting generalisability beyond specific disadvantaged rural settings [11–13,15,17]. Second, mental health outcomes were often assessed using non-validated, single-item measures, raising concerns about measurement validity and comparability [13,15]. Third, women’s empowerment was frequently captured using a narrow definition–commonly limited to decision-making power [13,15], thus failing to reflect its multidimensional nature. Fourth, findings regarding decision-making power have been inconsistent; while several studies report a protective association [12–15], others found that greater decision-making autonomy was associated with increased risk of depression and anxiety [10,17].

Additionally, despite the growing scholarly interest in this topic within South Asia, such as India [12] and Nepal [10], the mental health implications of women’s empowerment have been critically understudied in the context of Bangladesh. Notably, several studies have begun to document the burden of mental health problems among Bangladeshi women, underscoring the high risk of common mental disorders in this population [18–21]. However, these studies have largely overlooked the structural gender inequalities that underlie and exacerbate such conditions [22,23]. Bangladesh represents one of the most deeply patriarchal societies in South Asia, where entrenched gender norms and cultural expectations continue to shape women’s everyday lives [24]. According to the nationally representative Bangladesh Violence Against Women Survey 2024, 75.9% of ever-married women reported experiencing intimate partner violence (IPV) in their lifetime, and 48.7% within the past 12 months [25]. Female labour force participation rate stands at 44.2%, largely concentrated in the livestock sector and garment manufacturing, compared with 80.9% among men, who are widely represented in construction, transportation, and large-scale services [26]. Economic disparities persist, with a gender gap in bank account ownership of 19.4%, a figure exceeding the South Asia regional average [27]. Autonomy within the household also remains limited, with only 44% of women participating in all types of household decision-making [28]. These persistent gender inequalities are often deeply interlinked with religious and cultural norms in Bangladesh. Prior research suggests that patriarchal interpretations of religious beliefs and social customs may reinforce expectations that women’s roles remain primarily within the family sphere, thereby limiting women’s opportunities for education, employment, and participation in public life [24,29].

Given the persistence of patriarchal structures in Bangladesh, there is a critical need to examine whether women’s empowerment serves as a protective factor against mental distress or whether disempowerment exacerbates their depression and anxiety symptoms. The current study sought to address this gap by using the most recent nationally representative data in Bangladesh, which includes comprehensive information on women’s empowerment among currently married women as well as widely recognised and validated screening tools of depression and anxiety. Specifically, we employed the globally validated survey-based Women’s emPowERment index (SWPER), which assesses women’s empowerment across three domains [30]: attitude to violence, indicating whether women justify wife-beating; social independence, indicating women’s preconditions to be self-sufficient (*e.g.* education, age at marriage), and decision-making, indicating women’s participation in household decisions. By identifying how specific domains of empowerment associate with mental health among currently married women, this study seeks to inform the design of mental health strategies that not only support married women’s psychological well-being but also address underlying gendered barriers in the Bangladeshi context.

## METHODS

### Data and study population

In this study, we adhered to all items outlined in GRABDROP guidelines [31] (Table S1 in the **Online Supplementary Document**).

We utilised the most recent nationally representative data from the 2022 Bangladesh Demographic and Health Survey (DHS). The DHS are standardised household surveys conducted across LMICs that collect comprehensive data on a wide range of public health indicators, including maternal and child health, as well as emerging chronic conditions such as hypertension and mental health disorders. Detailed information on the DHS sampling design and data collection procedures is available elsewhere [32].

**Figure 1:**
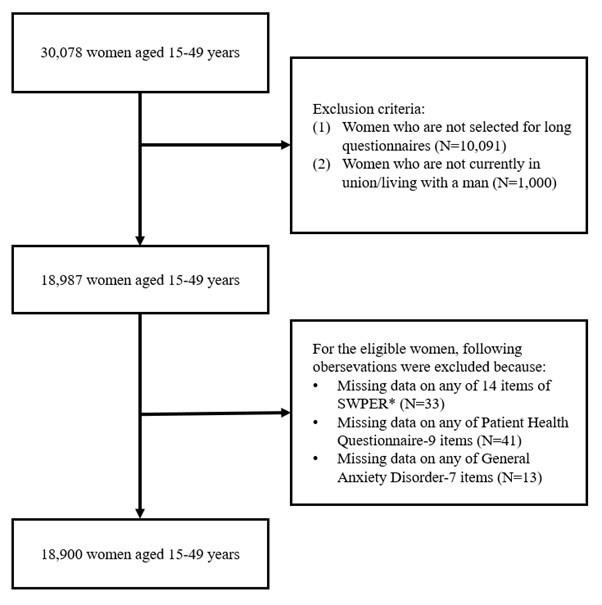
Flow diagram of the study sample derived from the 2022 Bangladesh Demographic and Health Surveys. SWPER – Survey-based Women’s emPowERment index.

The 2022 Bangladesh DHS initially surveyed 30 078 women aged 15–49 years. Mental health variables were collected through the DHS long questionnaire, which in Bangladesh was administered to a randomly selected two-thirds subsample of households as part of the survey design [33]. Therefore, women from households not selected for the long questionnaire (n = 10 091) were excluded from the present analysis. In addition, we excluded women who were not currently in a union (n = 1000), as current marital status was a prerequisite for most items in the SWPER index, resulting in 18 987 eligible women. We further excluded women with missing data on any of the 14 SWPER items (n = 33), the Patient Health Questionnaire-9 (PHQ-9) items used to measure depressive symptoms (n = 41), and the Generalised Anxiety Disorder-7 (GAD-7) items used to measure anxiety symptoms (n = 13). Of note, item-level missingness in the PHQ-9 and GAD-7 items was minimal, with an average of 3.3 missing responses per item across the analytic sample. The final analytic sample consisted of 18 900 women (**Figure 1**).

This study was exempt from ethical approval (KUIRB-2023-0245-01) as it utilised secondary analysis of publicly available data. We also adhered to the STROBE reporting guidelines (Table S2 in the **Online Supplementary Document**).

### Measures

#### Exposure variables

We employed the SWPER index, a widely used and globally validated measure well-known for capturing the multidimensional nature of women’s empowerment [30]. SWPER consists of three key domains: attitudes toward intimate partner violence, social independence, and decision-making. The attitude to violence domain includes five items capturing women’s perceptions of whether a husband is justified in hitting his wife under specific circumstances (*e.g.* if she goes out without informing him, or if she argues with him). The social independence domain includes six items reflecting enabling resources for empowerment, such as a woman’s level of education, age at first marriage, age at first birth, and access to media. The decision-making domain includes three items evaluating women’s participation in household decisions, including those related to women’s health care and large household purchases.

Consistent with the methodology outlined by Ewerling and colleagues [30], we extracted the relevant 14 items and calculated domain scores using the published SWPER global scoring coefficients. For each domain, higher scores indicate higher levels of women’s empowerment (Table S3 in the **Online Supplementary Document**). The resulting standardised scores were subsequently categorised into terciles (low, medium, high) for each domain, following previously established SWPER-based studies [34,35].

#### Outcome variables

Depressive symptoms were measured using the PHQ-9, a widely used self-report tool based on the Diagnostic and Statistical Manual of Mental Disorders criteria for assessing the presence of depressive symptoms over the past two weeks. The PHQ-9 has demonstrated good reliability and factorial validity in Bangladeshi populations [36], supporting its use in this context. In the 2022 Bangladesh DHS, each item was rated on a 0–3 scale using the validated Water Glass Pictorial Scale, with response categories corresponding to ‘0’ (never), ‘1’ (rarely), ‘2’ (often), and ‘3′ (always) [33]. Internal consistency in our analytic sample demonstrated good reliability (Cronbach’s α = 0.81). We then summarised the item scores to generate a total PHQ-9 score ranging from zero to 27. Severity was categorised as follows: none (0–4), mild (5–9), moderate (10–14), moderately severe (15–19), and severe (20–27). Following the cutoff suggested by the original validation study [37], a score ≥10 was used as the threshold to indicate the presence of depressive symptoms (coded as ‘1’), while scores <10 indicated the absence (coded as ‘0’).

We assessed anxiety symptoms using the GAD-7 scale, a brief instrument evaluating the frequency and intensity of anxiety symptoms over the past two weeks. Evidence from Bangladeshi populations indicates that the GAD-7 exhibits robust psychometric properties, including reliable internal consistency and stable factor structure [38]. Similar to the PHQ-9, response options were coded as ‘0’ (never), ‘1’ (rarely), ‘2’ (often), and ‘3′ (always) using the Water Glass Pictorial Scale [33]. Internal consistency in our analytic sample was also good (Cronbach’s α = 0.83). The total score, ranging from zero to 21, was obtained by summing all responses. Severity was as follows: none (0–4), mild (5–9), moderate (10–14), and severe (15–21). Consistent with a prior validation study [39], a score ≥10 was used to indicate clinically relevant anxiety symptoms (coded as ‘1’), whereas scores <10 were considered to indicate no symptoms (coded as ‘0’).

### Sociodemographic covariates

Women’s age (15–19, 20–29, 30–39, and 40–49 years) and education (no education, primary, secondary, and higher) were adjusted for in the attitude to violence and decision-making models, as they were considered potential confounders of the associations with mental health outcomes. In contrast, these variables were not included in the social independence models because they are intrinsic components of that domain. All models further adjusted for women’s occupation (not working/housewife, agricultural, professional/technical/skilled, and services/others), husband’s type of occupation (same categories with women’s occupation), number of child aged <5 years (none, one, two, and ≥3), number of household members (one to 25), sex of household head (male and female), place of residence (urban and rural), household wealth index (poorest, poorer, middle, richer, and richest), religion (Islam, Hindu, Buddhist, and others), and Division (Barishal, Chattogram, Dhaka, Khulna, Mymensingh, Rajshahi, Rangpur, and Sylhet).

### Statistical analysis

First, we conducted a descriptive analysis to characterise the study population and explore patterns of mental health outcomes across women’s empowerment levels. Weighted percentages were used to describe the distribution of sociodemographic variables and prevalence of mental health outcomes across empowerment levels. We then estimated the prevalence of depressive and anxiety symptoms across terciles of each SWPER domain (attitude to violence, social independence, and decision-making) and assessed group differences using Pearson χ^2^ tests.

Second, we fitted a series of multivariable logistic regression models to examine the association between women’s empowerment and mental health outcomes. For each mental health outcome, separate models were specified for each SWPER domain, with empowerment levels entered as categorical variables in terciles (low, medium, high). All models adjusted for relevant sociodemographic covariates. To further explore potential heterogeneity in symptom severity, we conducted additional analyses disaggregating outcomes within the clinically established threshold (≥10). Depressive symptoms were categorised as moderate (PHQ-9 score 10–14) and severe (15–27), and anxiety symptoms as moderate (GAD-7 score 10–14) and severe (15–21), following established severity classifications [37,40].

Finally, we conducted a sensitivity analysis to assess the robustness of our main findings by treating the SWPER domain scores as continuous variables rather than categorical terciles, in order to examine whether the observed associations were sensitive to the specification of the exposure.

All analyses accounted for the DHS’s complex multistage sampling design. We applied the women’s individual sampling weights (v005) in accordance with DHS guidelines. Clusters were specified using variable ‘v021’, and stratification was accounted for using variable ‘v023.’ We implemented survey design adjustments using Stata’s ‘svyset’ command, and all descriptive and regression analyses were conducted with the ‘svy’ prefix to obtain design-corrected standard errors and confidence intervals. All regression models additionally included division fixed effects to account for potential regional heterogeneity across administrative divisions in Bangladesh.

We performed analyses using Stata MP, version 16.1 (StataCorp LLC, College Station, Texas, USA). Adjusted odds ratios (aORs) with 95% confidence intervals (CIs) were reported. We considered a *P*-value <0.05 statistically significant.

## RESULTS

Among the 18 900 currently married women in Bangladesh, the majority were not working or housewives (65.1%), belonged to male-headed households (87.3%), resided in rural areas (71.7%), and were Muslims (90.4%) (**Table 1**).

**Table 1:** Sociodemographic characteristics among married women in Bangladesh (n = 18 900)

Characteristics	n (%)*
Age group	
*15–19*	1598 (8.9)
*20–29*	6551 (34.8)
*30–39*	6459 (34.1)
*40–49*	4292 (22.3)
Educational level	
*No education*	2426 (13.0)
*Primary*	4864 (25.7)
*Secondary*	8765 (47.4)
*Higher*	2845 (13.9)
Women’s occupation	
*Not working/housewife*	12 599 (65.1)
*Agricultural*	3798 (21.4)
*Professional/technical/skilled*	1438 (7.6)
*Services/others*	1065 (6.0)
Husband’s occupation	
*Not working/housewife*	714 (3.7)
*Agricultural*	4389 (23.9)
*Professional/technical/skilled*	6924 (35.8)
*Services/others*	6873 (36.7)
Number of children aged <5 y	
*0*	9729 (51.1)
*1*	7074 (38.0)
*2*	1753 (9.1)
*>3*	344 (1.8)
Number of household members, x̄ (SD)	5.05 (2.3)
Sex of household head	
*Male*	16 616 (87.3)
*Female*	2284 (12.7)
Place of residence	
*Urban*	6595 (28.3)
*Rural*	12 305 (71.7)
Wealth index	
*Poorest*	3338 (17.6)
*Poorer*	3707 (20.2)
*Middle*	3765 (20.6)
*Richer*	3926 (21.0)
*Richest*	4164 (20.6)
Religion	
*Islam*	16 937 (90.4)
*Hindu*	1746 (8.3)
*Buddhist*	175 (1.1)
*Others*	42 (0.2)
Division	
*Barishal*	2025 (6.1)
*Chattogram*	2802 (18.6)
*Dhaka*	2860 (25.3)
*Khulna*	2462 (11.9)
*Mymensingh*	2043 (7.6)
*Rajshahi*	2430 (13.2)
*Rangpur*	2288 (11.5)
*Yylhet*	1990 (5.7)

SD – standard deviation, x̄ – mean

*Individual women’s weights were applied for the prevalence of sociodemographic characteristics.

Of 18 900 currently married Bangladeshi women, 4.6% reported depressive symptoms, and 4.0% had anxiety symptoms (**Table 2**). In both the attitude to violence and social independence domains, women with low empowerment consistently exhibited the highest prevalence of depressive and anxiety symptoms (*P* < 0.05). For example, in the attitude to violence domain, the prevalence of depressive symptoms was 5.8% among women with low empowerment, compared to 3.4% among women with high empowerment. Meanwhile, in the decision-making domain, women with medium empowerment showed the highest prevalence of both mental health outcomes, with comparable prevalence rates observed among women with high and low empowerment.

**Table 2:** Prevalence of mental health outcomes by women’s empowerment level among married women in Bangladesh (n = 18 900)

Items	Depressive symptoms*		Anxiety symptoms†	
	**n (%)‡**	**OR (95% CI)§**	**n (%)‡**	**OR (95% CI)§**
	**899 (4.6)**		**760 (4.0)**	
Attitude to violence				
*High*	216 (3.4)	Ref.	188 (2.9)	Ref.
*Medium*	298 (4.5)	1.21 (0.97–1.51)	240 (3.8)	1.12 (0.88–1.41)
*Low*	385 (5.8)	1.48 (1.17–1.86)¶	332 (5.2)	1.41 (1.09–1.82)¶
Social independence				
*High*	265 (4.0)	Ref.	222 (3.6)	Ref.
*Medium*	296 (4.5)	1.10 (0.90–1.35)	224 (3.4)	0.90 (0.73–1.12)
*Low*	338 (5.3)	1.22 (0.99–1.50)	314 (4.9)	1.28 (1.02–1.60)║
Decision-making				
*High*	285 (4.4)	Ref.	234 (3.6)	Ref.
*Medium*	355 (5.1)	1.03 (0.84–1.26)	322 (4.8)	1.12 (0.91–1.39)
*Low*	259 (4.3)	0.91 (0.72–1.15)	204 (3.5)	0.90 (0.68–1.17)

CI – confidence interval, OR – odds ratio, ref – reference

*Depressive symptoms were defined as having moderate to severe depression, indicated by a PHQ-9 score ≥10.

†Anxiety symptoms were defined as having moderate to severe anxiety, indicated by a GAD-7 score ≥10.

‡Individual women's weights were applied for the prevalence of mental health outcomes.

§Separate models were specified for each SWPER domain for each mental health outcome, accounting for the complex multistage sampling design of the DHS. Covariates included women’s occupation (not working/housewife, agricultural, professional/technical/skilled, and services/others), husband’s type of occupation (same categories with women’s occupation), number of child aged <5 y (0, 1, 2, and ≥3), number of household members (1–25), sex of household head (male and female), place of residence (urban and rural), household wealth index (poorest, poorer, middle, richer, and richest), religion (Islam, Hindu, Buddhist, and others), and Division (Barishal, Chattogram, Dhaka, Khulna, Mymensingh, Rajshahi, Rangpur, and Sylhet). Models for the attitude to violence and decision-making domains were additionally adjusted for women’s age (15–19, 20–29, 30–39, and 40–49 y) and education (no education, primary, secondary, and higher).

¶*P* < 0.01.

║*P* < 0.05.

In the attitude to violence domain, women with low empowerment were 1.48 times more likely to experience depressive symptoms (aOR = 1.48; 95% CI = 1.17–1.86) and 1.41 times more likely to have anxiety symptoms (aOR = 1.41; 95% CI = 1.09–1.82) compared to women with high empowerment, after adjusting sociodemographic covariates including women’s age, education, occupation, religion, and household wealth (**Table 2**). Medium empowerment was not significantly associated with higher odds of depression or anxiety. In the social independence domain, women with low empowerment were 1.28 times more likely to experience anxiety symptoms (aOR = 1.28; 95% CI = 1.02–1.60) compared to women with high empowerment, after accounting for other covariates. Women with low empowerment were also 1.22 times more likely to have depressive symptoms (aOR = 1.22; 95% CI = 0.99–1.50), but the association was not statistically significant. In the decision-making domain, neither medium nor low empowerment was significantly associated with depressive or anxiety symptoms after adjusting for covariates.

When disaggregated by symptom severity (**Table 3**), low empowerment in the attitude to violence domain was significantly associated with moderate depressive symptoms (aOR = 1.66; 95% CI = 1.31–2.11) and moderate anxiety symptoms (aOR = 1.35; 95% CI = 1.05–1.72), compared with high empowerment. However, no statistically significant associations were observed for severe depression or severe anxiety in this domain. In the social independence domain, low empowerment was significantly associated with moderate anxiety symptoms (aOR = 1.40; 95% CI = 1.14–1.73), whereas its association with moderate depressive symptoms was not statistically significant. No significant associations were found for severe outcomes. In contrast, the decision-making domain showed the opposite pattern for severe outcomes. Low empowerment was associated with lower odds of severe depressive symptoms (aOR = 0.48; 95% CI = 0.33–0.70) and severe anxiety symptoms (aOR = 0.44; 95% CI = 0.28–0.71), while no significant associations were observed for moderate outcomes.

**Table 3:** Multivariable associations between each domain of women’s empowerment and severity of mental health outcomes among married women in Bangladesh (n = 18 900)

Items	Depressive symptoms*				Anxiety symptoms†			
	**Moderate**		**Severe**		**Moderate**		**Severe**	
	**n (%)‡**	**OR (95% CI)§**	**n (%)‡**	**OR (95% CI)§**	**n (%)‡**	**OR (95% CI)§**	**n (%)‡**	**OR (95% CI)§**
Attitude to violence								
*High*	155 (2.5)	Ref.	61 (0.9)	Ref.	152 (2.3)	Ref.	36 (0.5)	Ref.
*Medium*	228 (3.4)	1.37 (1.10–1.72)ǁ	70 (1.2)	0.89 (0.61–1.29)	182 (2.9)	0.99 (0.78–1.25)	58 (0.9)	1.12 (0.71–1.77)
*Low*	292 (4.5)	1.66 (1.31–2.11)¶	93 (1.3)	1.09 (0.73–1.63)	263 (4.2)	1.35 (1.05–1.72)**	68 (0.9)	1.16 (0.71–1.90)
Social independence								
*High*	196 (2.9)	Ref.	69 (1.0)	Ref.	171 (2.8)	Ref.	51 (0.8)	Ref.
*Medium*	224 (3.4)	1.11 (0.91–1.36)	72 (1.1)	1.04 (0.74–1.47)	173 (2.6)	1.00 (0.80–1.24)	51 (0.8)	0.94 (0.63–1.39)
*Low*	255 (4.0)	1.16 (0.95–1.41)	83 (1.3)	1.21 (0.86–1.69)	254 (4.1)	1.40 (1.14–1.73)ǁ	60 (0.9)	1.05 (0.70–1.56)
Decision-making								
*High*	199 (3.1)	Ref.	86 (1.3)	Ref.	176 (2.7)	Ref.	58 (1.0)	Ref.
*Medium*	266 (3.8)	1.20 (0.99–1.47)	89 (1.3)	0.81 (0.58–1.12)	248 (3.8)	1.22 (0.99–1.51)	74 (1.0)	0.95 (0.65–1.39)
*Low*	310 (3.5)	1.01 (0.82–1.24)	49 (0.8)	0.48 (0.33–0.70)¶	174 (3.0)	0.94 (0.75–1.18)	30 (0.4)	0.44 (0.28–0.71)ǁ

CI – confidence interval, OR – odds ratio, ref – reference

*Depressive symptom severity was categorised as moderate (PHQ-9 score 10–14) and severe (PHQ-9 score 15–27).

†Anxiety symptom severity was categorised as moderate (GAD-7 score 10–14) and severe (PHQ-9 score 15–21).

‡Individual women’s weights were applied for the prevalence of mental health outcomes.

§Separate models were specified for each SWPER domain and each mental health outcome, accounting for the DHS’s complex multistage sampling design. Covariates included women’s occupation (not working/housewife, agricultural, professional/technical/skilled, and services/others), husband’s type of occupation (same categories with women’s occupation), number of child aged <5 y (0, 1, 2, and ≥3), number of household members (1–25), sex of household head (male and female), place of residence (urban and rural), household wealth index (poorest, poorer, middle, richer, and richest), reli­gion (Islam, Hindu, Buddhist, and others), and Division (Barishal, Chattogram, Dhaka, Khulna, Mymensingh, Rajshahi, Rangpur, and Sylhet). Models for the attitude-to-violence and decision-making domains were additionally adjusted for women’s age (15–19, 20–29, 30–39, and 40–49 y) and education (no education, primary, secondary, and higher).

¶*P* < 0.001.

║*P* < 0.01.

**P < 0.05.

Consistent with the primary analysis, the sensitivity analysis yielded largely similar patterns of association. When the SWPER domain scores were modelled as continuous variables, the overall trends remained unchanged (Table S4 in the **Online Supplementary Document**). Specifically, the associations between the attitude to violence domain and both depression and anxiety remained statistically significant, while minor changes in effect sizes and statistical significance were observed for the social independence domain.

## DISCUSSION

Using the 2022 nationally representative data set, this study found that a considerable proportion of currently married women in Bangladesh experience depression (4.6%) and anxiety (4.0%). These figures represent a slight increase from 2019 estimates reported by the Global Burden of Disease study, which reported the prevalence of major depressive disorders and anxiety disorders in Bangladesh at 3.5% and 3.2%, respectively [4]. As one of the few population-level estimates available from LMICs using nationally representative data, our study provides timely and context-specific evidence on the mental health status of women in Bangladesh, one of the most patriarchal societies in South Asia where women’s mental health has long been understudied. Yet, our findings should be interpreted with caution, as the reported prevalence may be underestimating the true burden due to underreporting driven by persistent mental health stigma and low mental health literacy in the country [18].

Most importantly, women’s empowerment emerged as a significant factor associated with mental health outcomes even after adjusting for various sociodemographic covariates, consistent with prior literature in South Asia [10,41,42], although the magnitude and direction of associations varied across domains. The attitude to violence showed the strongest and most consistent relationship with both mental health outcomes. In the social independence domain, women’s low empowerment was significantly associated with anxiety, but not with depression. In the decision-making domain, no significant associations were found with either depression or anxiety. When disaggregated by symptom severity, the attitude to violence and social independence domains exhibited patterns that were similar to the overall findings for moderate symptoms. In contrast, the decision-making domain showed a different pattern: low empowerment was associated with lower odds of severe depressive and anxiety symptoms, indicating an inverse relationship that diverged from the direction observed in the other domains.

Low empowerment in the attitude to violence domain, marked by strong justification of IPV [30], reflects a range of psychosocial and structural vulnerabilities that may account for its significant association with depression and anxiety. First, and most directly, women who justify IPV may be at increased risk of experiencing it. In a study of Uganda, women’s acceptance of gender norms justifying wife-beating increased their likelihood of experiencing sexual, emotional, and physical IPV [43]. Numerous studies have documented a strong association between IPV exposure and poor mental health outcomes, including elevated risks of depression, anxiety, posttraumatic stress disorder, and suicidality [44,45]. Second, women’s justification or acceptance of IPV highly suggests that they are more likely to internalise blame and suppress emotional responses to chronic adversity, including fear, anger, and shame. Prior studies have shown that this pattern of self-blame, shame, and emotional suppression is closely associated with depression and anxiety [46]. Third, justifying IPV may also reflect a broader mistrust in institutional protection mechanisms. In many patriarchal settings, women’s experiences of violence are often dismissed or disbelieved by law enforcement or judicial systems, discouraging them from reporting abuse or seeking help [47]. Studies have noted that women often ‘pass off’ violence as a normal event because prior help-seeking attempts were met with indifference or disbelief [47]. This accumulated sense of helplessness and institutional betrayal may further erode mental well-being.

The significant association between women’s low empowerment and anxiety – but not depression – in the social independence domain deserves nuanced interpretation. This domain, as conceptualised in the SWPER, comprises women’s preconditions for self-sufficient lives, including educational attainment, age at first marriage, media exposure, and spousal age disparity [30]. Limited access to education and media may curtail women’s mental health literacy, impeding recognition of psychological distress, help-seeking behaviours, and use of adaptive coping strategies for ongoing stressors [48], all leading to increased anxiety. A recent study of Iranian adults, for example, revealed that low socioeconomic status (*e.g.* lower educational level) led to lower health literacy, which again contributed to poor mental health [16]. Likewise, women’s early marriage and substantial age gaps between spouses may heighten financial dependence and power asymmetries within spousal relationships [49]. Such conditions can foster a persistent sense of uncertainty, vigilance, and worry – maladaptive responses that are more proximally associated with anxiety than depression [50]. Our findings imply that deficits in structural and informational resources may not necessarily lead to the emotional flattening or affective numbing characteristics of depression, but rather exacerbate over-arousal, hypervigilance, and internal tension that underpin anxiety disorders. Further longitudinal and qualitative studies are needed to elucidate why women’s low social independence is associated with anxiety but not depression, and to uncover the psychological and causal pathways underlying this divergence.

Finally, the pattern observed in the decision-making domain warrants careful interpretation. In contrast to the other domains, decision-making empowerment was not significantly associated with overall depressive or anxiety symptoms. However, when disaggregated by severity, low empowerment was associated with lower odds of severe depressive and anxiety symptoms, suggesting that women with higher levels of decision-making power may bear a disproportionate burden of severe mental health symptoms. Although counterintuitive, this finding echoes several studies in LMIC suggesting that women’s greater household decision-making power increases the risk of poor mental health [10,11,19,51]. A study in rural Burkina Faso, for instance, found that women’s decision-making authority over credit was significantly associated with higher levels of depression [11]. Similarly, research in Uzbekistan reported that women with greater decision-making autonomy were more likely to experience depression [51]. From this perspective, higher levels of decision-making authority may reflect not only empowerment but also role expansion within persistent patriarchal structures. In Bangladeshi settings where caregiving and domestic roles remain largely unchanged, greater participation in household decisions may entail expanded responsibilities, economic pressure, and conflict negotiation without corresponding support, potentially increasing vulnerability to severe psychological strain [19].

At the same time, the lower risk of mental health problems observed for women with low decision-making power may be partly reflected in limitations in how decision-making is operationalised within the SWPER index. The index assigns equal weight to independent and joint decision-making, which may not adequately capture perceived autonomy in highly gender-unequal settings where independent decisions may reflect role obligations rather than genuine agency. As such, the seemingly protective association among women with low decision-making power should be interpreted cautiously, as measurement constraints may have obscured underlying dynamics. Future work should further disentangle the complex interplay between women’s perceived agency, responsibility, and intra-household gender power dynamics, including unpacking how different types of decisions shape women’s mental health.

In Bangladesh, substantial structural challenges persist in the mental health care system, including a lack of public mental health facilities, a shortage of trained professionals, and inadequate financial investment [52,53]. For example, the country allocates less than 0.5% of its health budget to mental health care [52], and over 64% of total health expenditure comes from out-of-pocket payments [53], exacerbating inequities and limiting access for the most vulnerable population. These systemic barriers are further compounded for women by deeply entrenched social and cultural stigma surrounding mental disorders. Within Bangladesh’s highly patriarchal context, women were often labelled as emotionally fragile or unfit mothers, with fears of losing child custody or social standing, which deterred them from seeking help. Family members, including husbands and parents, frequently encouraged women to conceal their symptoms, and some women have even discontinued therapy due to judgmental attitudes from health professionals [23]. Given that women’s low empowerment in their acceptance of violence and lack of social independence was strongly associated with poor mental health in our study, the overlapping barriers of gendered stigma and societal pressure that women should remain in the domestic sphere may pose a critical obstacle in women’s mental health trajectories. Without addressing the normative environment that marginalises women’s psychological distress and restricts their care-seeking, efforts to improve mental health among Bangladeshi women are likely to fall short. Therefore, beyond expanding service availability, gender-transformative approaches that actively challenge discriminatory norms and inequitable gender roles, reduce women’s disproportionate care responsibilities, and promote women’s social standing are essential to creating a supportive environment for women’s mental well-being.

Specifically, policymakers should recognise the gendered nature of psychological distress [54] and develop empowerment-oriented mental health strategies that reflect women’s lived realities. A strong example of such an approach is the ‘Moms’ Empowerment Program,’ a community-based group intervention implemented in the USA for Latina mothers who have experienced IPV [55]. Sessions included safety planning, identification of community resources, and strategies to build social capital, thereby helping participants recognise and navigate structural barriers while strengthening their agency. Participation in this program has been associated with significant reductions in depression and posttraumatic stress, as well as improvements in positive parenting. At the same time, health system reforms should include gender-responsive design, such as recruiting more female health care professionals, ensuring safe, stigma-free service environments, and embedding gender-sensitive and empowerment frameworks into the training curricula of all mental health and primary care providers [56]. These measures can help mitigate gendered access barriers, build trust with women service users, and support the broader goal of transforming health systems into enablers of equity and empowerment. Finally, our findings highlight the need for a validated composite measure of women’s empowerment that integrates multiple domains into a single index. While our domain-specific approach offers conceptual clarity, a composite measure could facilitate policy and programmatic relevance by enabling more efficient resource allocation, improving service planning and delivery, and supporting consistent monitoring across settings and cross-country comparisons. Ultimately, addressing both structural gender inequities in access to care and limitations of current measurement frameworks is essential to advancing women’s mental health in a comprehensive and sustainable way.

A few key limitations should be acknowledged. First, the cross-sectional design hinders causal inference regarding the relationship between women’s empowerment and mental health outcomes. Residual confounding may also exist due to previous mental health conditions, ongoing psychosocial stressors, or social support networks, all of which were unavailable in the current data. Additionally, we cannot rule out the potential reverse causation since women’s mental distress can influence their empowerment process. Nonetheless, this study provides valuable insights by utilising the most recent nationally representative data in Bangladesh to estimate national-level prevalence among currently married women. Future research employing longitudinal or mixed-method designs is needed to better examine the complex pathways linking women’s empowerment and mental health, including potential mechanisms such as exposure to gender-based violence, levels of social support, and access to economic resources. Second, the findings are not generalisable to the entire female population, as the SWPER index applies only to women who are currently married or in union. Descriptive comparisons suggested that non-married women tended to show a stronger socioeconomic gradient in depressive and anxiety symptoms, particularly among older and poorer groups. Because these characteristics were also associated with lower empowerment (data not shown), excluding these women could slightly underestimate the associations examined in this study. However, given the relatively small number of non-married women, the overall impact on the estimates is likely limited. Third, the SWPER does not capture all facets of women’s empowerment – particularly economic, legal, and political domains. Moreover, the decision-making domain of SWPER relies on who usually makes household decisions, which may not adequately reflect the depth of women’s autonomy. Fourth, although SWPER scores were categorised into terciles for interpretability and comparability with prior studies, these cutoffs represent rank-based groupings and may introduce artificial divisions in the underlying distribution. Yet, the SWPER is a highly recommended measure in LMICs for its global validation and multidimensionality. Fifth, as formal clinical diagnostic validation studies of specific thresholds remain limited in Bangladesh, we applied the widely used PHQ-9 and GAD-7 cutoffs (≥10) derived from the original validation studies. Future research should evaluate culturally and clinically appropriate thresholds for depression and anxiety screening in the Bangladeshi context. Lastly, our study focuses on individual-level associations and does not model contextual determinants. However, analyses incorporating cluster fixed effects yielded largely similar results to the main models (data not shown). Future research should examine contextual determinants (*e.g.* community norms, health service availability, and urbanisation) that may shape women’s mental health.

## CONCLUSIONS

This study provides timely, population-based evidence that women’s empowerment is significantly associated with mental health outcomes among currently married women in Bangladesh, a country marked by entrenched gender inequalities. Our findings highlight that women’s low empowerment – particularly in the attitude to violence – elevates the risk of depression and anxiety. Addressing women’s mental health requires more than expanding clinical services; it demands a structural and normative shift that promotes women’s social standing and challenges patriarchal norms. Importantly, women’s empowerment should be recognised as a multi-dimensional factor associated with health, not only influencing mental well-being but also shaping access to and utilisation of broader health and nutrition services. In this context, the Sustainable Development Goals three (good health and well-being) and five (gender equality) should not be approached in isolation. Rather, they are deeply interconnected, and progress in one can generate synergistic benefits in the other. Investing in women’s empowerment is not only a matter of human rights but also a strategic imperative to improve women’s mental health. Achieving meaningful gains in mental health in LMICs settings thus calls for integrated, gender-transformative strategies that advance both equity and well-being.

## Additional material


Online Supplementary Document
