# Supplementary material for: Exploring the link between women’s empowerment, depression, and anxiety in Bangladesh: evidence from a nationally representative cross-sectional study
Source: J Glob Health. 2026 May 29;16:04189. doi: 10.7189/jogh.16.04189 (PMC13220647; doi:10.7189/jogh.16.04189)
Supplement: Online Supplementary Document [file jogh-16-04189-s001.pdf]

Supplement to: Eom YJ, Jang S, Hasman A, Subramanian SV, Kim R. Exploring the link between women's empowerment, depression, and anxiety in Bangladesh: evidence from a nationally representative cross-sectional study. J Glob Health. 2026;16:04189.

Table S1. Adherence to JoGH's GRABDROP guidelines items

JoGH guideline items
1. Please list all papers published by each co-author in previous 3 years that were based on secondary analysis of a big data repository

Yun-Jung Eom

- Eom YJ, Balla S, Rajpal S, Kim R, Subramanian SV. Assessing geographic variation in women's decision-making power across 720 districts in India, 2016–2021. BMC Women's Health. 2025;26(1).
- Gausman J, Eom YJ, Kim R, Subramanian SV. Small area geographic variation in girl and boy child marriage in India: a district-level longitudinal analysis, 2016 and 2021. Journal of Global Health. 2025;15.
- Eom YJ, Subramanian SV, Kim R. Geographic variation in women's empowerment: a multilevel analysis of India's National Family Health Survey 2021. Journal of global health. 2025;15:04159.
- Chi H, Eom YJ, Jeong J, Lee HY, Kim R. Joint parental stimulation and early childhood development in 26 sub-Saharan African countries. BMJ Paediatrics Open. 2025;9(1):e003091–1.
- Eom YJ, Chi H, Bhatia A, Lee HY, Subramanian SV, Kim R. Individual- and community-based women's empowerment and complete use of maternal healthcare services: a multilevel analysis of 34 sub-Saharan African countries. Social Science & Medicine. 2025;117816–6.
- Kim J, Eom YJ, Ko S, Subramanian SV, Kim R. Problems accessing health care and under-5 mortality: a pooled analysis of 50 low- and middle-income countries. Journal of Public Health. 2024;46(3):315–25.
- Eom YJ, Chi H, Jung S, Kim J, Jeong J, Subramanian SV, et al. Women's empowerment and child anthropometric failures in 28 sub-Saharan African countries: A cross-level Interaction by gender inequality index. SSM - Population Healt . 2024;101651.
- Jung S, Chi H, Eom YJ, Subramanian SV, Kim R. Multilevel analysis of determinants in postnatal care utilisation among mother-newborn pairs in India, 2019–21. Journal of Global Health. 2024;14.
- Chi H, Eom YJ, Jung S, Kim J, Jeong J, Kim R. Maternal Decision-Making Power and Care-Seeking Behaviors for Acutely Ill Children: A Multilevel Analysis of 33 Sub-Saharan African Countries. The American Journal of Tropical Medicine and Hygiene. 2024;110(2):370–8.

Sion Jang

- Jang S. The Effect of Overtime Work on Young Wage Workers' Depressive Symptom - Analysis of the Mediating Effect of Work-Life Imbalance. Korean Journal of Social Welfare. 2025;77(2):291-317. [In Korean]
- Jang S, Lee J. Predictive Factors of Suicidal Ideation among Single-Household Elderly who Experienced Spouse Bereavement: Using Logistic Regression. Korean Journal of Gerontological Social Welfare. 2025;80(2):93-122. [In Korean]
- Jang S. Does Stress Affect Adolescent Anxiety by Mediating Sleep Time? Stress. 2024;32(3):142-152. [In Korean]
- Jang S, Oh E. The Effect of Cultural Adaptation Stress on School Life Satisfaction of Multicultural Adolescents: An Analysis of the Moderating Effect of Visible and Invisible Appearance Characteristics Based on the Concept of Visible Minority. Korean Journal of Youth Welfare. 2024;26(4):51-81. [In Korean]
- Jang S, Kim D. The Moderating Effect of Social Support on the Impact of Drinking Frequency on Young Adults' Depression: Analysis of Gender Difference. Health and Social Welfare Review. 2024;44(1):191-217. [In Korean]

- Jang S. The Effect Relationship between Cyber Violence Victimization and Adolescent's Mental Health Vulnerability: Group Comparative Analysis by Type of Victim-Perpetrator Relationship. Journal of Korean Criminological Association. 2024;18(3):67-95. [In Korean]
- Jang S. Analysis of Occupational Safety Hazard Factors that Hinder Health and Medical Worker's Mental Well-being - Focusing on Doctor, Nurse, Medical Technician. Health and Social Science. 2024;66:51-87. [In Korean]
- Jang S, Oh E. Impact of 5060 generation middle-aged people's experience of unmet healthcare on life dissatisfaction: Verification of the mediating effect of depression. Korean Journal of Health Education and Promotion. 2024;41(3):33-48. [In Korean]
- Lee S, Lim J, Jang S, Oh Y. A Study of the Impact of Work Environment on Depressive Symptoms in Older Security Guards. Korean Journal of Gerontological Social Welfare. 2023;78(3):167-194. [In Korean]
- Jang S, Kim M. The Effect of Depression on Adolescent Delinquent Behavior : Focusing on Companionship among Peer Group. Korean Journal of Youth Studies. 2023;30(6):31-59. [In Korean]

Andreas Hasman

- Koivu A, Ashorn U, Borghi E, Hasman A, Menon P, Pulungan A, et al. Global assessment of childhood growth monitoring: cross-sectional survey of national policies and practices. Journal of Global Health. 2026;16:04034.
- Corball L, Chappel K, Rowett H, Laillou A, Hasman A, Kawai N. Cost-Effectiveness Analysis of Vitamin A Supplementation Delivery Modalities in DRC, Togo, and Niger: Informing Sustainable Program Design. Health Policy and Planning. 2026;czag032.
- Tang K, Eilerts H, Imohe A, Adams KP, Sandalinas F, Moloney G, et al. Evaluating equity dimensions of infant and child vitamin A supplementation programmes using Demographic and Health Surveys from 49 countries. BMJ Open. 2023;13(3):e062387.
- Nonvignon J, Aryeetey G,, Adjagba A, Asman J, Sharkey A, Hasman A, Pallas S, Griffiths U. The political economy of financing traditional vaccines and vitamin A supplements in six African countries. Health Policy and Planning. 2023;38(10):1154-1165.
- Karlsson O, Kim R, Moloney G, Hasman A, Subramanian SV. Patterns in child stunting by age: A cross-sectional study of 94 low- and middle-income countries. Maternal and Child Nutrition. 2023;19(4):e13537.

S V Subramanian

- Pal A, Kim R, Subramanian SV. Prevalence of widowhood across states and union territories in India, 1993–2021: a repeated cross-sectional study. J Glob Health. 2026;16:04068.
- Sadhu R, Ko S, Subramanian SV, Kim R. Everyday discrimination among middle-aged and older adults in India: a multilevel cross-sectional analysis from the Longitudinal Ageing Study in India. Sci Rep. 2026.
- Kalita A, Zadey S, Shukla SK, Bhadada S, Kane S, Roy D, et al. The Citizens Survey 2022–23: a household-level dataset on Universal Health Coverage in India. Sci Data. 2026.
- Balla S, Kim R, Saunik S, Subramanian SV. Problems in accessing healthcare among women in India: a district-level change analysis, 2016–2021. BMC Public Health. 2026;26:544.
- Longkumer I, Ko S, DeSouza P, Bhatia R, Kim R, Subramanian SV. Polluting cooking fuels and life satisfaction among middle-aged and older adults: a cross-sectional study from the Longitudinal Ageing Study in India. GeroScience. 2026:1–14.
- Patel V, Kalita A, Furtado KM, Mor N, Bhadada S, Albert S, et al. The Lancet Commission on a citizen-centred health system for India. Lancet. 2026;407(10526):388–468.

- Chi H, Puno A, Jain A, Subramanian SV, Kim R. Type of water and sanitation facilities and risk for non-partner sexual violence: a multilevel analysis across 31 low- and middle-income countries. Soc Sci Med. 2026:119005.
- Park D, Kim R, Shin MJ, Saunik S, Subramanian SV. Geographic variation in lack of food group consumption among children in India: an analysis of change across 720 districts, 2016–2021. PLOS Glob Public Health. 2026;6(1):e0005077.
- Sung M, Subramanian SV, Kim R. The gender distribution and association between sociodemographic factors and hospital-presenting self-injury: analysis from the Korea National Hospital Discharge In-Depth Injury Survey. Arch Suicide Res. 2026;30(1):131–149.
- Shah A, Kim R, Subramanian SV. Heat, health and inequality: access to cooling equipment in India. J Dev Policy Pract. 2026.
- DeSouza P, Lee J, Longkumer I, Kar A, Nemeth J, Al-Kindi S, et al. Associations between the transition to cleaner cooking energy use and child health outcomes in India. SSRN. 2026.
- Jain A, Kim R, Subramanian SV. Assessing geographic variations in household water disruptions across 30,109 communities in India in 2021. PLOS Water. 2026;5(1):e0000334.
- Pal A, Maiti S, Zhelenkova E, Kim R, Subramanian SV. Geographic inequalities in employment indicators among men and women in India: an analysis of 720 districts, 2019–2021. J Dev Policy Pract. 2026.
- Jain A, Kumar A, Kim R, Subramanian SV. Prevalence and burden of no-toilet households in India: an analysis of 261,746 households in 36 states/Union Territories in 2022–2023. Glob Health Action. 2025;18(1):2511351.
- Eom YJ, Balla S, Rajpal S, Kim R, Subramanian SV. Assessing geographic variation in women’s decision-making power across 720 districts in India, 2016–2021. BMC Womens Health. 2025.
- Jain A, Adamkiewicz G, Kim R, Subramanian SV. Changes in housing quality across and within India’s 720 districts between 2016 and 2021. SSM Popul Health. 2025:101899.
- Beyer L, Blossom J, Chen J, Kim R, Subramanian SV. Life expectancies across school districts in the United States. SSM Popul Health. 2025:101896.
- Park S, Dieleman JL, Kim R, Subramanian SV. Association of health and social spending with health outcomes in OECD countries. Health Serv Res. 2025;60(6):e14660.
- DeSouza P, Lee JJ, Németh J, Mani S, Jain A, Kar A, Peel J, Al-Kindi S, et al. Evaluating associations between the transition to cleaner cooking energy use and hypertension in India. Environ Res Health. 2025;3(4):045008.
- Rai RK, Bromage S, Bayan B, Ratha BC, Kim R, Dubey SK, et al. Relationship between fish consumption and undernutrition among young Indian children. Curr Dev Nutr. 2025:107610.
- Rai RK, Bromage S, Bayan B, Ratha BC, Kim R, Dubey SK, et al. Fish consumption and its association with anaemia and metabolic disorder among Indian adults. Br J Nutr. 2025:1–13.
- Rajpal S, Ronanki S, Sathesh N, Kim R, Subramanian SV. Trends in nulliparous singleton alive births by cesarean section in India: empirical patterns across public and private hospitals for 720 districts, 2016–2021. PLOS Glob Public Health. 2025;5(11):e0005501.
- Novak D, Emeljanovas A, Kim R, Ko S, Subramanian SV. School-level variation in physical fitness outcomes among children and adolescents. Sci Rep. 2025.
- Bogler L, Kumar A, Subramanian SV, Vollmer S. Effects of a large-scale participatory learning and action programme in women’s groups on knowledge and behaviour related to pregnancy and childcare: a cluster-randomized trial. SSM Popul Health. 2025:101880.
- Shah A, Sugathan A, Malghan D, Kim R, Subramanian SV. Spatiotemporal changes in heat stress exposure in India, 1981–2023. Nat Commun. 2025;16(1):9496.
- Leckie G, Bell A, Merlo J, Subramanian SV, Evans C. The statistical advantages of multilevel analysis of individual heterogeneity and discriminatory accuracy for estimating intersectional inequalities. Sociol Methods Res. 2025.

- Kwon KY, Jang H, Subramanian SV, Kim J. School-based social relationships and children's psychological health: examining heterogeneity by relationship source and child gender. Eur Child Adolesc Psychiatry. 2025.
- Sadhu R, Kim R, Subramanian SV, Danaei G. Religious group differences in non-communicable disease risk factors in India: a cross-sectional study of adults aged 45 and older. GeroScience. 2025.
- Fuller-Rowell TE, Sultana S, Ryff CD, Kim ES, Jokela M, Subramanian SV, et al. Childhood SES gradients in adult functional limitations: does state-level macro-economic context matter? Soc Sci Med. 2025:118676.
- Gausman J, Eom YJ, Kim R, Subramanian SV. Small area geographic variation in girl and boy child marriage in India: a district-level longitudinal analysis, 2016 and 2021. J Glob Health. 2025;15:04248.
- Jain A, Ambade M, Balla S, Pal A, Rajpal S, Kim R, Subramanian SV. Measuring changes in prevalence of hypertension and diabetes across 720 districts in India using cross-sectional data from 2016 to 2021. BMJ Public Health. 2025;3(2).
- Matsuoka Y, Yoshida H, Kawachi I, Subramanian SV, Sawada Y, et al. Can proximity to blue spaces and cooling centers buffer extreme heat impacts on walking steps? Eur J Public Health. 2025;35(Suppl 4).
- Ambade M, Kim R, Subramanian SV. Socioeconomic and geographic patterns of cost for latest hospitalization and outpatient service use among older adults aged 45 years and over in India. In: Handbook of Aging, Health and Public Policy: Perspectives from Asia. 2025:1825–1844.
- Rajpal S, Ko S, Leckie G, Jain D, Blossom JC, Kim R, Subramanian SV. India policy insights: estimates of population health and social determinants indicators across policy units. Sci Data. 2025;12(1):1592.
- Park D, Lee DH, Kim R, Shin MJ, Subramanian SV. Prevalence of clinical obesity in US adults based on a newly proposed definition. JAMA Netw Open. 2025;8(9):e2533806.
- Jain A, Kim R, Subramanian SV. Prevalence and treatment of diarrhea among children in India, 2016–2021. JAMA Netw Open. 2025;8(8):e2526979.
- Leckie G, Bell A, Merlo J, Subramanian SV, Evans C. The statistical advantages of MAIHDA for estimating intersectional inequalities. Sociol Methods Res. 2025.
- Kalita A, Zadey S, Shukla SK, Bhadada S, Kane S, Roy D, et al. The citizens survey on healthcare in India. 2025.
- Jain A, Kim R, Subramanian SV. Analyzing changes in types of household sanitation among 543 parliamentary constituencies between 2016 and 2021 in India. PLOS Water. 2025;4(8):e0000409.
- Park D, Shin MJ, Subramanian SV, Park CY, Kim R. Individual- and neighborhood-level factors influencing diet quality: a multilevel analysis using Korea National Health and Nutrition Examination Survey data, 2010–2019. Epidemiol Health. 2025;47:e2025043.
- Son H, Jang H, Subramanian SV, Kim J. Physical disabilities in adolescence and educational attainment in adulthood: the mediating role of expectations, discipline, and school relationships. Youth Soc. 2025.
- Liao J, Sung M, Kim R, Subramanian SV. Testing Geoffrey Rose's assumption of uniform population change for body mass index distribution in India. Lancet Reg Health Southeast Asia. 2025.
- Kephart LL, Rees VW, Giovenco DP, Subramanian SV. The unequal geography of recreational cannabis retailers in the US. Am J Prev Med. 2025;69(2):107643.
- Novak D, Oršolić M, Barbaros P, Suzuki E, Subramanian SV. Family ties and social capital among grand slam tennis champions. Int Rev Sociol Sport. 2025;60(5):900–920.
- Bogler L, Kumar A, Subramanian SV, Vollmer S. Effects of a participatory learning and action programme in women's groups on adolescent girls: a cluster-randomized controlled trial in Bihar, India. SSM Popul Health. 2025:101840.
- Rajpal S, Joe W, Ronanki S, Kim R, Subramanian SV. Centenarians and near-centenarians in India: empirical insights on health and well-being characteristics. GeroScience. 2025:1–13.

- Jain A, Pitchik HO, Kim R, Subramanian SV, Glymour MM. Examining the association between toilet access and major depression among older adults in India. Age Ageing. 2025;54(7):afaf170.
- Sommer ML, Bogler L, Subramanian SV, Vollmer S. The impact of a participatory learning and action intervention on unmet need for contraception: a cluster-randomized controlled trial in rural Bihar, India. Reprod Health. 2025;22(1):121.
- Eom YJ, Subramanian SV, Kim R. Geographic variation in women's empowerment: a multilevel analysis of India's National Family Health Survey 2021. J Glob Health. 2025;15:04159.
- Liao J, Kumar A, Kim R, Subramanian SV. Disability among school children across districts of India. JAMA Netw Open. 2025;8(6):e2517223.
- Karlsson O, Kumar A, Kim R, Subramanian SV. Trends in low birth weight across 36 states and union territories in India, 1993–2021. BMJ Glob Health. 2025;10(6).
- Park S, Dieleman J, Kim R, Subramanian SV. Association of health and social spending with disability-adjusted life years and death among 37 OECD countries between 2000 and 2019: a fixed effects analysis. 2025 Annual Research Meeting. 2025.
- Oke I, Geanacopoulos AT, Subramanian SV, Wu AC, Bardach NS, et al. State-level policies and antibiotic use for pediatric conjunctivitis. JAMA Pediatr. 2025;179(6):679-681.
- Kim J, Choe SA, Lee HY, Subramanian SV, Kim R. Rural-urban migration dynamics and double burden of malnutrition among women across 29 low and middle income countries. Soc Sci Med. 2025;374:118047.
- Yang L, Subramanian SV. Is the dual process model of bereavement effective among older bereaved parents in China? An examination of the oscillation process and the effects of overloading. Soc Sci Med. 2025;374:118042.
- Rajpal S, Kumar A, Ronanki S, Sathesh N, Kim R, Subramanian SV. Changes in prevalence of alcohol and tobacco consumption across districts of India, 2016 and 2021. BMC Public Health. 2025;25(1):1962.
- Subramanian S, Kumar A, Pullum TW, Ambade M, Rajpal S, Kim R. Early-neonatal, late-neonatal, postneonatal, and child mortality rates across India, 1993–2021. JAMA Netw Open. 2025;8(5).
- Joe W, Prakash A, Ahluwalia K, Kim R, Subramanian SV. Association between risk of mortality among children and twin birth in India: an econometric analysis of live births between 1993–2021. J Glob Health. 2025;15:04136.
- Jain A, Kumar A, Pullum TW, Kim R, Swaminathan S, Subramanian SV. Trends in socioeconomic inequality in mortality during childhood between 1993 and 2021 in India. BMJ Glob Health. 2025;10(5).
- Jain D, Kachinovsky J, Rodriguez G, Chen J, Kim R, Subramanian SV. India Policy Insights: a geospatial and temporal data science and visualization platform and architecture. SoftwareX. 2025;30:102149.
- Karlsson O, Pullum TW, Kumar A, Kim R, Subramanian SV. Age decomposition of mortality rates among children younger than 5 years in 47 LMICs. JAMA Pediatr. 2025;179(5):540-549.
- Ko S, Puno-Balagosa A, Rajpal S, Joe W, Ramanathan M, et al. Women's working status and intimate partner violence victimization in India: the role of husbands' attitudes toward violence. J Fam Violence. 2025:1-12.
- Liao J, Moshoeshoe R, Holmes MD, Subramanian SV, De Neve JW. Effect of girls' education on cancer awareness and screening in a natural experiment in Lesotho. Nat Commun. 2025;16(1):3737.
- Pradhan J, Pai M, Dwivedi R, Mishra B, Behera S, Tera B, Kim R, et al. Burden of non-communicable diseases in South Asia: a decomposition analysis. J Health Popul Nutr. 2025;44(1):124.
- Zhang Y, Tong G, Ma N, Chen S, Kong Y, Rahmartani LD, Aheto JMK, et al. Associations between education and ideal cardiovascular health metrics across 36 low- and middle-income countries. BMC Med. 2025;23(1):204.

- Karlsson O, Rajpal S, Johri M, Kim R, Subramanian S. Prevalence and trends of not receiving a dose of DPT-containing vaccine among children 12-35 months: an analysis of 81 low- and middle-income countries (vol 14, pg 1490, 2024). J Epidemiol Glob Health. 2025;15(1).
- Karlsson O, Rajpal S, Johri M, Kim R, Subramanian SV. Correction: Prevalence and trends of not receiving a dose of DPT-containing vaccine among children 12–35 months: an analysis of 81 low- and middle-income countries. J Epidemiol Glob Health. 2025;15(1):52.
- Zhao S, Liu S, Gao J, Ma N, Chen S, Chandan JS, Kim R, Karoli P, Niyi JL, et al. Prevalence of co-occurring forms of intimate partner violence against women aged 15–49 and the role of education-related inequalities: analysis of Demographic and Health Surveys. EClinicalMedicine. 2025;82.
- Eom YJ, Chi H, Bhatia A, Lee HY, Subramanian SV, Kim R. Individual- and community-level women's empowerment and complete use of maternal healthcare services: a multilevel analysis of 34 sub-Saharan African countries. Soc Sci Med. 2025;370:117816.
- Son H, Jang H, Park H, Subramanian SV, Kim J. Exploring the trajectories of depressive symptoms associated with bullying victimization: the intersection of gender and family support. J Adolesc. 2025;97(3):746-757.
- Narayanan M, Karlsson O, Kumar A, Pullum TW, Kim R, Subramanian SV. Prevalence of severe and moderate anthropometric failure among children in India, 1993–2021. Matern Child Nutr. 2025;21(2):e13751.
- Chen S, Ma N, Kong Y, Chen Z, Niyi JL, Karoli P, Msuya HM, Zemene MA, et al. Prevalence, disparities, and trends in intimate partner violence against women living in urban slums in 34 low-income and middle-income countries: a multi-country cross-sectional analysis. EClinicalMedicine. 2025;81.
- Sadhu R, Kim R, Subramanian SV. Severe food insecurity among middle-aged and older adults in India: insights from the Longitudinal Aging Study in India. Glob Food Secur. 2025;44:100822.
- Jung S, Lee HY, Choe S, Oh H, Subramanian SV, Kim R. Maternal media exposure and child anthropometric failures across 40 low- and middle-income countries. SSM Popul Health. 2025;29:101746.
- Ko S, Oh H, Subramanian SV, Kim R. Life course social mobility and cognitive function among middle-aged and older adults in India: exploring heterogeneity by gender. Soc Sci Med. 2025;366:117640.
- Novak D, Čule M, Kim J, Kim R, Subramanian SV. Individual versus group exercise effect on youth physical activity levels: a randomised controlled trial. BMJ Open Sport Exerc Med. 2025;11(1).
- Novak D, Petrušič T, Čule M, Milinović I, Kim J, Kim R, Subramanian SV. Building social capital in university students: a physical education intervention program. J Phys Act Health. 2025;22(2):270-280.
- Bolsinger H, Subramanian S, Sinha M. AI in customer contexts: developing an analysis framework for trustful and responsible AI-integration. 2025.
- Jain A, Kim R, Subramanian SV. Analyzing changes in types of household sanitation among 543 Parliamentary Constituencies between 2016 and 2021 in India. PLOS Water. 2025;4(8):e0000409.
- Shah A, Kim R, Subramanian SV. Spatiotemporal changes in district-level carbon emissions in India, 2019–2024. Glob Sustain. 2025;8:e21.
- Jain A, Subramanian SV. Metrics for access to water and sanitation need structural changes. Lancet Glob Health. 2025;13(1):e12-e13.
- Kephart L, Rees VW, Subramanian SV, Giovenco DP. Exploring the association between neighborhood disadvantage and cannabis retail density: a multi-measure analysis. Health Place. 2025;91:103396.
- Johri M, Rajpal S, Kim R, Subramanian SV. Small-area variation in child under-vaccination in India: a multilevel analysis of cross-sectional data from 36 states and Union Territories, 707 districts, and 22,349 small areas. Lancet Reg Health Southeast Asia. 2025;32.
- Karlsson O, Rajpal S, Johri M, Kim R, Subramanian SV. Prevalence and trends of not receiving a dose of DPT-containing vaccine among children 12–35 months: an analysis of 81 low- and middle-income countries. J Epidemiol Glob Health. 2024;14(4):1490-1503.

- Karlsson O, Benski C, Kapoor M, Kim R, Subramanian SV. Association between neonatal mortality and births not weighed among 400 thousand institutional deliveries in 32 low- and middle-income countries. J Public Health. 2024;46(4):e614-e622.
- Ambade M, Kim R, Subramanian SV. Experience of health care utilization for inpatient and outpatient services among older adults in India. Public Health Pract. 2024;8:100541.
- Lippert AM, Corsi DJ, Kim R, Wedow R, Kim J, Taddess B, et al. Polygenic and socioeconomic contributions to nicotine use and cardiometabolic health in early mid-life. Nicotine Tob Res. 2024;26(12):1616-1625.
- Karlsson O, Kim R, Subramanian SV. International trends in zinc treatment for diarrhea. Pediatrics. 2024;154(5):e2024066701.
- Jang H, Son H, Subramanian SV, Kim J. The spillover of violence: the gendered relationship between parental physical violence and peers' bullying victimization. Child Youth Serv Rev. 2024;166:107978.
- Lee HY, Kumar A, Jain A, Kim R, Subramanian SV. Trends in the quality of antenatal care in India: patterns of change across 36 states and union territories, 1999–2021. J Glob Health. 2024;14:04188.
- Jain A, Rajpal S, Rana MJ, Kim R, Subramanian SV. Correction: Small area variations in four measures of poverty among Indian households: econometric analysis of National Family Health Survey 2019–2021. Humanit Soc Sci Commun. 2024;11(1):1-17.
- Sung M, Jain A, Kumar A, Kim R, Kulkarni B, Subramanian SV. Patterns of change in the association between socioeconomic status and body mass index distribution in India, 1999–2021. J Glob Health. 2024;14:04171.
- Ambade M, Mishra R, Kim R, Subramanian SV. Association between 23 correlates anthropometric failure among children: analysis of 2016 and 2021 National Family Health Surveys in India. Coll Antropol. 2024;48(3):197-224.
- Bansal A, Dwivedi LK, Bhatia M, Subramanian SV. Inter-generational impact: exploring the influence of older sister-in-law's contraceptive choices on her peer contraception adoption in India. Sex Reprod Healthc. 2024;41:101004.
- Kim J, Eom YJ, Ko S, Subramanian SV, Kim R. Problems accessing health care and under-5 mortality: a pooled analysis of 50 low- and middle-income countries. J Public Health. 2024;46(3):315-325.
- Boing AC, Boing AF, Borges ME, Rodrigues DO, Barberia L, et al. Spatial clusters and social inequities in COVID-19 vaccine coverage among children in Brazil. Cien Saude Colet. 2024;29:e03952023.
- Jain A, Harrison C, Kumar A, Kim R, Subramanian SV. Examining geographic variation in the prevalence of household drainage types across India in 2019–2021. NPJ Clean Water. 2024;7(1):71.
- Shapiro DJ, Geanacopoulos AT, Subramanian SV, Wu AC, Bardach NS, et al. Antibiotic treatment and health care use in children and adolescents with conjunctivitis. JAMA Ophthalmol. 2024;142(8):779-780.
- Jain A, Kim R, Swaminathan S, Subramanian SV. Socioeconomic inequality in child health outcomes in India: analyzing trends between 1993 and 2021. Int J Equity Health. 2024;23(1):149.
- Mukherji A, Rao M, Desai S, Subramanian SV, Kang G, Patel V. District-level monitoring of universal health coverage, India. Bull World Health Organ. 2024;102(9):630.
- Boing AF, Boing AC, Barberia L, Borges ME, Subramanian SV. Correction: The Brazilian vaccine divide: how some municipalities were left behind in the Covid-19 vaccine coverage. PLOS Glob Public Health. 2024;4(6):e0003447.
- Evans CR, Borrell LN, Bell A, Holman D, Subramanian SV, Leckie G. Clarifications on the intersectional MAIHDA approach: a conceptual guide and response to Wilkes and Karimi (2024). Soc Sci Med. 2024;350:116898.
- Evans CR, Leckie G, Subramanian SV, Bell A, Merlo J. A tutorial for conducting intersectional multilevel analysis of individual heterogeneity and discriminatory accuracy (MAIHDA). SSM Popul Health. 2024;26:101664.

- Eom YJ, Chi H, Jung S, Kim J, Jeong J, Subramanian SV, Kim R. Women's empowerment and child anthropometric failures across 28 sub-Saharan African countries: a cross-level interaction by gender inequality index. SSM Popul Health. 2024;26:101651.
- Sung M, Kumar A, Mishra R, Kulkarni B, Kim R, Subramanian SV. Temporal change in prevalence of BMI categories in India: patterns across states and Union territories of India, 1999–2021. BMC Public Health. 2024;24(1):1322.
- Subramanian SV, Kumar A, Pullum TW, Ambade M, Rajpal S, Kim R. Early-neonatal, late-neonatal, postneonatal, and child mortality rates across India, 1993–2021. JAMA Netw Open. 2024;7(5):e2410046.
- Jung S, Chi H, Eom YJ, Subramanian SV, Kim R. Multilevel analysis of determinants in postnatal care utilisation among mother-newborn pairs in India, 2019–21. J Glob Health. 2024;14:04085.
- Chi H, Jung S, Subramanian SV, Kim R. Socioeconomic and geographic inequalities in antenatal and postnatal care components in India, 2016–2021. Sci Rep. 2024;14(1):10221.
- Dholakia A, Burdick KJ, Kreatsoulas C, Monuteaux MC, Tsai J, et al. Historical redlining and present-day nonsuicide firearm fatalities. Ann Intern Med. 2024;177(5):592-597.
- Earnshaw VA, Sepucha KR, Laurenceau JP, Subramanian SV, Hill EC, et al. Disclosing Recovery: a pilot randomized controlled trial of a patient decision aid to improve disclosure processes for people in treatment for opioid use disorder. J Subst Use Addict Treat. 2024;160:209291.
- Devaraj K, Gausman J, Mishra R, Kumar A, Kim R, Subramanian SV. Trends in prevalence of unmet need for family planning in India: patterns of change across 36 states and Union territories, 1993–2021. Reprod Health. 2024;21(1):48.
- Bogler L, Bommer C, Ebert C, Kumar A, Subramanian SV, et al. Effects of a large-scale participatory learning and action programme in women's groups on health, nutrition, water, sanitation, and hygiene: a cluster-randomised controlled trial. J Dev Effect. 2024;16(2):246-263.
- Kephart L, Rees V, Giovenco D, Subramanian SV. Cannabis retail density and concentrated neighborhood advantage and disadvantage: a comparison of neighborhood differences using multiple measures of density. In: Making "Weed Maps": Using Mapping to Evaluate (in) Equity in Cannabis Retail … 2024.
- Kephart L, Rees V, Giovenco D, Subramanian SV. The proximity and density of alcohol and tobacco outlets near recreational cannabis retailers in legalized states: assessing neighborhood disparities in the retail environment. In: Making "Weed Maps": Using Mapping to Evaluate (in) Equity in Cannabis Retail … 2024.
- Kephart L, Rees V, Giovenco D, Subramanian SV. The location of recreational cannabis retailers and structural neighborhood advantage and disadvantage in the United States. In: Making "Weed Maps": Using Mapping to Evaluate (in) Equity in Cannabis Retail … 2024.
- Kapoor M, Ambade M, Ravi S, Subramanian SV. Age- and gender-specific prevalence of intellectually disabled population in India. J Autism Dev Disord. 2024;54(4):1594-1604.
- Sung M, Kim R, Subramanian SV. The gender distribution and association between sociodemographic factors and self-injury: analysis from the Discharge Injury Patient Survey, South Korea. 2024.
- Lv R, Huang Y, Huang S, Wu S, Wang S, Hu G, Ma Y, Song P, et al. Associations between parental adherence to healthy lifestyles and cognitive performance in offspring: a prospective cohort study in China. Chin Med J (Engl). 2024;137(06):683-693.
- Jain A, Sharma S, Kim R, Subramanian SV. Food deprivation among adults in India: an analysis of specific food-categories, 2016–2021 (vol 66, 102313, 2023). EClinicalMedicine. 2024;69.
- Subramanian SV, Patnaik A, Kim R. Call for action: presenting constituency-level data on population, health and socioeconomic wellbeing related to 2030 Sustainable Development Goals for India. Lancet Reg Health Southeast Asia. 2024;22.
- Jain A, Sharma S, Kim R, Subramanian SV. Corrigendum to—"Food deprivation among adults in India: an analysis of specific food-categories, 2016–2021" [EClinicalMedicine 66 (2023) 102313]. EClinicalMedicine. 2024;69.

- Wang H, Chen Z, Li Z, He X, Subramanian SV. How economic development affects healthcare access for people with disabilities: a multilevel study in China. SSM Popul Health. 2024;25:101594.
- Ambade M, Menon N, Subramanian SV. The impact of early-life access to oral polio vaccines on disability: evidence from India. J Popul Econ. 2024;37(1):23.
- Karlsson O, Kim R, Subramanian SV. Prevalence of children aged 6 to 23 months who did not consume animal milk, formula, or solid or semisolid food during the last 24 hours across low- and middle-income countries. JAMA Netw Open. 2024;7(2):e2355465.
- Chen Z, Sharma S, Chen S, Kim R, Subramanian SV, Li Z. Prevalence, trend, and inequality of prolonged exclusive breastfeeding among children aged 6–23 months old in India from 1992–2021: a cross-sectional study of nationally representative data. J Glob Health. 2024;14:04026.
- Gausman J, Kim R, Kumar A, Ravi S, Subramanian SV. Prevalence of girl and boy child marriage across states and Union Territories in India, 1993–2021: a repeated cross-sectional study. Lancet Glob Health. 2024;12(2):e271-e281.
- Ko S, Park S, Kim J, Subramanian SV, Kim R. Spousal multimorbidity and depressive symptoms among older Indian couples: do one's own health status and sex matter? GeroScience. 2024;46(1):885-896.
- Boing AC, Boing AF, Borges ME, Rodrigues DO, Barberia L, et al. Aglomerados espaciais e desigualdades sociais na cobertura vacinal contra COVID-19 em crianças no Brasil. Cien Saude Colet. 2024;29:e03952023.
- Ravi S, Kapoor M, Rajpal S, Subramanian SV. Change in religious composition across districts in India from 2001 to 2011: a descriptive analysis of the religion census. Econ Polit Wkly. 2024;59(10):55.
- Kim HJ, Cho S, Shin SY, Lee SK, Kim H, Srinivasan S, Subramanian SV, et al. Traffic exposure and breast cancer mortality by area of residence: incorporating clinical and socioeconomic data. In: MEDINFO 2023—The Future Is Accessible. 2024:1566-1567.
- Subramanian SV, Joe W. Population, health and nutrition profile of the Scheduled Tribes in India: a comparative perspective, 2016–2021. Lancet Reg Health Southeast Asia. 2024;20.
- Matsuki N, Suzuki E, Mitsuhashi T, Subramanian SV, Takao S, Yorifuji T. Complementary and alternative medicine use, cancer screening, and medical checkups in Japan from 2001 to 2013: a repeated cross-sectional study. J Integr Complement Med. 2024;30(1):47-56.
- Li Z, Wang H, Chen S, Kong Y, Xie L, Zhang X, Lu C, Subramanian SV, et al. The association of a disability-targeted cash transfer programme with disability status and health-care access: a quasi-experimental study using a nationwide cohort of 4.3 million people in China. Lancet Public Health. 2023;8(12):e933-e942.
- Jain A, Sharma S, Kim R, Subramanian SV. Food deprivation among adults in India: an analysis of specific food categories, 2016–2021. EClinicalMedicine. 2023;66.
- Ma N, Chen S, Kong Y, Chen Z, Geldsetzer P, Zeng H, Wu L, et al. Prevalence and changes of intimate partner violence against women aged 15 to 49 years in 53 low-income and middle-income countries from 2000 to 2021: a secondary analysis of Demographic and Health Surveys. Lancet Glob Health. 2023;11(12):e1863-e1873.
- Spoer BR, Chen AS, Lampe TM, Nelson IS, Vierse A, Zazanis NV, Kim B, et al. Validation of a geospatial aggregation method for congressional districts and other US administrative geographies. SSM Popul Health. 2023;24:101511.
- Johri M, Ng ESW, Sharkey A, Bosson-Rieutort D, Kone GK, et al. Effects of zero-dose vaccination status in early childhood and level of community socioeconomic development on learning attainment in preadolescence in India: a population-based study. BMJ Public Health. 2023;1(1).
- Boing AF, Boing AC, Barberia L, Borges ME, Subramanian SV. The Brazilian vaccine divide: how some municipalities were left behind in the Covid-19 vaccine coverage. PLOS Glob Public Health. 2023;3(11):e0002493.
- Ko S, Oh H, Subramanian SV, Kim R. Small area geographic estimates of cardiovascular disease risk factors in India. JAMA Netw Open. 2023;6(10):e2337171.
- deSouza PN, Chaudhary E, Dey S, Ko S, Németh J, Guttikunda S, et al. An environmental justice analysis of air pollution in India. Sci Rep. 2023;13(1):16690.
- Ambade M, Kim R, Subramanian SV. Socio-economic distribution of modifiable risk factors for cardiovascular diseases: an analysis of the national longitudinal ageing study in India. Prev Med. 2023;175:107696.

- Karlsson O, Kim R, Moloney GM, Hasman A, Subramanian SV. Patterns in child stunting by age: a cross-sectional study of 94 low- and middle-income countries. Matern Child Nutr. 2023;19(4):e13537.
- Dhamija G, Kapoor M, Kim R, Subramanian SV. Explaining the poor-rich gap in anthropometric failure among children in India: an econometric analysis of the NFHS, 2021 and 2016. SSM Popul Health. 2023;23:101482.
- Sinkovic F, Novak D, Foretic N, Kim J, Subramanian SV. The plyometric treatment effects on change of direction speed and reactive agility in young tennis players: a randomized controlled trial. Front Physiol. 2023;14:1226831.
- Klemm R, Hasman A, Karlsson O, Kim RR, Subramanian SV. Age distribution of all-cause mortality among children younger than 5 years: possible implications for VAS programs. Ann Nutr Metab. 2023;79:158-158.
- Madani A, Krause B, Greene ER, Subramanian S, Mohr BP, Holton JM, et al. Large language models generate functional protein sequences across diverse families. Nat Biotechnol. 2023;41(8):1099-1106.
- Kumari N, Saikia N, Subramanian SV. The puzzle of underreporting disability among tribal population in India: is it a statistical artifact or reality? GeroScience. 2023;45(4):2387-2403.
- Jain A, Kumar A, Kim R, Subramanian SV. Prevalence of zero-sanitation in India: patterns of change across the states and Union Territories, 1993–2021. J Glob Health. 2023;13:04082.
- Ferres JL, Nasir M, Bijral A, Subramanian SV, Weeks WB. Modeling to explore and challenge inherent assumptions when cultural norms have changed: a case study on left-handedness and life expectancy. Arch Public Health. 2023;81(1):137.
- Subramanian SV. Need for a structural approach to promote child survival. JAMA Netw Open. 2023;6(7):e2322435.
- Stein DT, Kim R, Subramanian SV, Bauhoff S. Geographic variation in healthcare utilization across villages, districts, and states in India: a multilevel modeling analysis. In: 2023 World Congress on Health Economics. 2023.
- Ambade M, Rajpal S, Kim R, Subramanian SV. Socioeconomic and geographic variation in coverage of health insurance across India. Front Public Health. 2023;11:1160088.
- Ravi S, Kapoor M, Subramanian SV. Assessing the National Surveys for its Representativeness. 2023.
- Subramanian SV, Ambade M, Sharma S, Kumar A, Kim R. Corrigendum to—"Prevalence of Zero-Food among infants and young children in India: patterns of change across the States and Union Territories of India, 1993–2021". EClinicalMedicine. 2023;61.
- Chatterjee P, Chen J, Yousafzai A, Kawachi I, Subramanian SV. When social identities intersect: understanding inequities in growth outcomes by religion-caste and religion-tribe as intersecting strata of social hierarchy for Muslim and Hindu children in India. Int J Equity Health. 2023;22(1):115.
- Boing AF, Boing AC, Barberia L, Borges ME, Subramanian SV. Uncovering inequities in Covid-19 vaccine coverage for adults and elderly in Brazil: a multilevel study of 2021–2022 data. Vaccine. 2023;41(26):3937-3945.
- Ko S, Kim R, Subramanian SV. Patterns in child health outcomes before and after the COVID-19 outbreak in India. JAMA Netw Open. 2023;6(6):e2317055.
- Novak D, Filip S, Subramanian SV. Developing an instrument to measure the social capital in young tennis players. Sport Mont. 2023;21(2):33-37.
- Karlsson O, Subramanian SV. Refrigerator ownership and child health and nutrition in low- and middle-income countries. Glob Food Secur. 2023;37:100698.
- Subramanian SV, Ambade M, Kumar A, Chi H, Joe W, Rajpal S, Kim R. Progress on Sustainable Development Goal indicators in 707 districts of India: a quantitative mid-line assessment using the National Family Health Surveys, 2016 and 2021. Lancet Reg Health Southeast Asia. 2023;13.
- Chatterjee P, Chen J, Yousafzai A, Kawachi I, Subramanian SV. Area level indirect exposure to extended conflicts and early childhood anthropometric outcomes in India: a repeat cross-sectional analysis. Confl Health. 2023;17(1):23.
- Subramanian SV, Ambade M, Sharma S, Kumar A, Kim R. Prevalence of Zero-Food among infants and young children in India: patterns of change across the States and Union Territories of India, 1993–2021. EClinicalMedicine. 2023;58.

- Park EH, Hwang S, Oh J, Kim BJ, Bae HJ, Yang KH, Choi AR, Kang MY, et al. Annual endovascular thrombectomy case volume and thrombectomy-capable hospitals of Korea in acute stroke care. J Prev Med Public Health. 2023;56(2):145.
- Jain A, Pitchik HO, Harrison C, Kim R, Subramanian SV. The association between anthropometric failure and toilet types: a cross-sectional study from India. Am J Trop Med Hyg. 2023;108(4):811.
- Rajpal S, Kumar A, Johri M, Kim R, Subramanian SV. Patterns in the prevalence of unvaccinated children across 36 states and Union Territories in India, 1993–2021. JAMA Netw Open. 2023;6(2):e2254919.
- Dwivedi LK, Bhatia M, Bansal A, Mishra R, P S, Jana S, Subramanian SV, et al. Role of seasonality variation in prevalence and trend of childhood wasting in India: an empirical analysis using National Family Health Surveys, 2005–2021. Health Sci Rep. 2023;6(2):e1093.
- Kim J, Park S, Subramanian SV, Kim T. The psychological costs of the COVID-19 pandemic and heterogeneous effects in South Korea: evidence from a difference-in-differences analysis. J Happiness Stud. 2023;24(2):455-476.
- Subramanian SV, Khailkar A, Karlsson O. Should India adopt a country-specific growth reference to measure undernutrition among its children? Lancet Reg Health Southeast Asia. 2023;9.
- Patnaik I, Sane R, Shah A, Subramanian SV. Distribution of self-reported health in India: the role of income and geography. PLoS One. 2023;18(1):e0279999.
- Chen S, Richardson S, Kong Y, Ma N, Zhao A, Song Y, Lu C, et al. Association between parental education and simultaneous malnutrition among parents and children in 45 low- and middle-income countries. JAMA Netw Open. 2023;6(1):e2251727.
- Jain A, Rajpal S, Rana MJ, Kim R, Subramanian SV. Small area variations in four measures of poverty among Indian households: econometric analysis of National Family Health Survey 2019–2021. Humanit Soc Sci Commun. 2023;10(1):18.

Rockli Kim

- Balla S, Kim R, Saunik S, Subramanian SV. Problems in accessing healthcare among women in India: a district-level change analysis, 2016–2021. BMC Public Health. 2026.
- Longkumer I, Ko S, deSouza P, Bhatia R, Kim R, Subramanian SV. Polluting cooking fuels and life satisfaction among middle-aged and older adults: a cross-sectional study from the Longitudinal Ageing Study in India. GeroScience. 2026:1-14.
- Chi H, Puno A, Jain A, Subramanian SV, Kim R. Type of water and sanitation facilities and risk for non-partner sexual violence: a multilevel analysis across 31 low- and middle-income countries. Soc Sci Med. 2026:119005.
- Park D, Kim R, Shin MJ, Saunik S, Subramanian SV. Geographic variation in lack of food group consumption among children in India: an analysis of change across 720 districts, 2016−2021. PLOS Glob Public Health. 2026;6(1):e0005077.
- DeSouza P, Lee J, Longkumer I, Kar A, Nemeth J, Al-Kindi S, Kumar P, et al. Associations between the transition to cleaner cooking energy use and child health outcomes in India. SSRN. 2026;6129466.
- Jain A, Kim R, Subramanian SV. Assessing geographic variations in household water disruptions across 30,109 communities in India in 2021. PLOS Water. 2026;5(1):e0000334.
- Pal A, Maiti S, Zhelenkova E, Kim R, Subramanian SV. Geographic inequalities in employment indicators among men and women in India: an analysis of 720 districts, 2019–2021. J Dev Policy Pract. 2026.
- Jain A, Kumar A, Kim R, Subramanian SV. Prevalence and burden of no-toilet households in India: an analysis of 261,746 households in 36 states/Union Territories in 2022–2023. Glob Health Action. 2025;18(1):2511351.
- Eom YJ, Balla S, Rajpal S, Kim R, Subramanian SV. Assessing geographic variation in women's decision-making power across 720 districts in India, 2016–2021. BMC Womens Health. 2025.

- Jain A, Adamkiewicz G, Kim R, Subramanian SV. Prevalence of housing structure and quality indicators in India: an assessment of changes across 720 districts between 2016 and 2021. SSM Popul Health. 2025;33:101899.
- Jain A, Adamkiewicz G, Kim R, Subramanian SV. Changes in housing quality across and within India's 720 districts between 2016 and 2021. SSM Popul Health. 2025:101899.
- Beyer L, Blossom J, Chen J, Kim R, Subramanian SV. Life expectancies across school districts in the United States. SSM Popul Health. 2025:101896.
- Park S, Dieleman JL, Kim R, Subramanian SV. Association of health and social spending with health outcomes in OECD countries. Health Serv Res. 2025;60(6):e14660.
- Rai RK, Bromage S, Bayan B, Ratha BC, Kim R, Dubey SK, et al. Relationship between fish consumption and undernutrition among young Indian children. Curr Dev Nutr. 2025:107610.
- Rai RK, Bromage S, Bayan B, Ratha BC, Kim R, Dubey SK, et al. Fish consumption and its association with anaemia and metabolic disorder among Indian adults. Br J Nutr. 2025:1-13.
- Rajpal S, Ronanki S, Sathesh N, Kim R, Subramanian SV. Trends in nulliparous singleton alive births by cesarean section in India: empirical patterns across public and private hospitals for 720 districts, 2016–2021. PLOS Glob Public Health. 2025;5(11):e0005501.
- Novak D, Emeljanovas A, Kim R, Ko S, Subramanian SV. School-level variation in physical fitness outcomes among children and adolescents. Sci Rep. 2025.
- DeSouza P, Lee JJ, Németh J, Mani S, Jain A, Kar A, Peel J, Al-Kindi S, et al. Evaluating associations between the transition to cleaner cooking energy use and hypertension in India. Environ Res Health. 2025;3(4):045008.
- Shah A, Sugathan A, Malghan D, Kim R, Subramanian SV. Spatiotemporal changes in heat stress exposure in India, 1981-2023. Nat Commun. 2025;16(1):9496.
- Sadhu R, Kim R, Subramanian SV, Danaei G. Religious group differences in non-communicable disease risk factors in India: a cross-sectional study of adults aged 45 and older. GeroScience. 2025:1-15.
- Gausman J, Eom YJ, Kim R, Subramanian SV. Small area geographic variation in girl and boy child marriage in India: a district-level longitudinal analysis, 2016 and 2021. J Glob Health. 2025;15:04248.
- Jain A, Ambade M, Balla S, Pal A, Rajpal S, Kim R, Subramanian SV. Measuring changes in prevalence of hypertension and diabetes across 720 districts in India using cross-sectional data from 2016 to 2021. BMJ Public Health. 2025;3(2).
- Kim R, Puno A, Choo S, Kim SS, Kim R. The association between victimization and perpetration of physical intimate partner violence and unmet healthcare needs among married women in South Korea. J Interpers Violence. 2025;40(19-20):4556-4573.
- Ambade M, Kim R, Subramanian SV. Socioeconomic and geographic patterns of cost for latest hospitalization and outpatient service use among older adults aged 45 years and over in India. In: Handbook of Aging, Health and Public Policy: Perspectives from Asia. 2025:1825-1844.
- Rajpal S, Ko S, Leckie G, Jain D, Blossom JC, Kim R, Subramanian SV. India Policy Insights: estimates of population health and social determinants indicators across policy units. Sci Data. 2025;12(1):1592.
- Park D, Lee DH, Kim R, Shin MJ, Subramanian SV. Prevalence of clinical obesity in US adults based on a newly proposed definition. JAMA Netw Open. 2025;8(9):e2533806.
- Jain A, Kim R, Subramanian SV. Prevalence and treatment of diarrhea among children in India, 2016-2021. JAMA Netw Open. 2025;8(8):e2526979.
- Jain A, Kim R, Subramanian SV. Analyzing changes in types of household sanitation among 543 Parliamentary Constituencies between 2016 and 2021. PLOS Water. 2025;4(8):e0000409.
- Park D, Shin MJ, Subramanian SV, Park CY, Kim R. Individual- and neighborhood-level factors influencing diet quality: a multilevel analysis using Korea National Health and Nutrition Examination Survey data, 2010-2019. Epidemiol Health. 2025;47:e2025043.

- Liao J, Sung M, Kim R, Subramanian SV. Testing Geoffrey Rose's assumption of uniform population change for body mass index distribution in India. Lancet Reg Health Southeast Asia. 2025;39.
- Rajpal S, Joe W, Ronanki S, Kim R, Subramanian SV. Centenarians and near-centenarians in India: empirical insights on health and well-being characteristics. GeroScience. 2025:1-13.
- Jain A, Pitchik HO, Kim R, Subramanian SV, Glymour MM. Examining the association between toilet access and major depression among older adults in India: a cross-sectional analysis of the Longitudinal Aging Study in India from 2017. Age Ageing. 2025;54(7):afaf170.
- Eom YJ, Subramanian SV, Kim R. Geographic variation in women's empowerment: a multilevel analysis of India's National Family Health Survey 2021. J Glob Health. 2025;15:04159.
- Liao J, Kumar A, Kim R, Subramanian SV. Disability among school children across districts of India. JAMA Netw Open. 2025;8(6):e2517223.
- Karlsson O, Kumar A, Kim R, Subramanian SV. Trends in low birth weight across 36 states and union territories in India, 1993-2021. BMJ Glob Health. 2025;10(6).
- Park S, Dieleman J, Kim R, Subramanian SV. Association of health and social spending with disability-adjusted life years and death among 37 OECD countries between 2000 and 2019: a fixed effects analysis. In: 2025 Annual Research Meeting. 2025.
- Sung M, Subramanian SV, Kim R. The gender distribution and association between sociodemographic factors and hospital-presenting self-injury: analysis from the Korea National Hospital Discharge In-Depth Injury Survey. Arch Suicide Res. 2025:1-19.
- Kim J, Choe SA, Lee HY, Subramanian SV, Kim R. Rural-urban migration dynamics and double burden of malnutrition among women across 29 low and middle income countries. Soc Sci Med. 2025;374:118047.
- Rajpal S, Kumar A, Ronanki S, Sathesh N, Kim R, Subramanian SV. Changes in prevalence of alcohol and tobacco consumption across districts of India, 2016 and 2021. BMC Public Health. 2025;25(1):1962.
- Subramanian S, Kumar A, Pullum TW, Ambade M, Rajpal S, Kim R. Early-neonatal, late-neonatal, postneonatal, and child mortality rates across India, 1993-2021 (vol 7, e2410046, 2024). JAMA Netw Open. 2025;8(5).
- Joe W, Prakash A, Ahluwalia K, Kim R, Subramanian SV. Association between risk of mortality among children and twin birth in India: an econometric analysis of live births between 1993–2021. J Glob Health. 2025;15:04136.
- Jain A, Kumar A, Pullum TW, Kim R, Swaminathan S, Subramanian SV. Trends in socioeconomic inequality in mortality during childhood between 1993 and 2021 in India. BMJ Glob Health. 2025;10(5).
- Jain D, Kachinovsky J, Rodriguez G, Chen J, Kim R, Subramanian SV. India Policy Insights: a geospatial and temporal data science and visualization platform and architecture. SoftwareX. 2025;30:102149.
- Puno-Balagosa A, Bhatia A, Jeong J, Kim R. Co-occurrence of maternal intimate partner violence and violent discipline and its associations with child morbidity in the Philippines. Child Abuse Negl. 2025;163:107363.
- Karlsson O, Pullum TW, Kumar A, Kim R, Subramanian SV. Age decomposition of mortality rates among children younger than 5 years in 47 LMICs. JAMA Pediatr. 2025;179(5):540-549.
- Ko S, Puno-Balagosa A, Rajpal S, Joe W, Ramanathan M, et al. Women's working status and intimate partner violence victimization in India: the role of husbands' attitudes toward violence. J Fam Violence. 2025:1-12.
- Pradhan J, Pai M, Dwivedi R, Mishra B, Behera S, Bera T, Kim R, et al. Burden of non-communicable diseases in South Asia: a decomposition analysis. J Health Popul Nutr. 2025;44(1):124.
- Chi H, Eom YJ, Jeong J, Lee HY, Kim R. Joint parental stimulation and early childhood development in 26 sub-Saharan African countries. BMJ Paediatr Open. 2025;9(1):e003091.

- Zhang Y, Tong G, Ma N, Chen S, Kong Y, Rahmartani LD, Aheto JMK, et al. Associations between education and ideal cardiovascular health metrics across 36 low- and middle-income countries. BMC Med. 2025;23(1):204.
- Karlsson O, Rajpal S, Johri M, Kim R, Subramanian S. Prevalence and trends of not receiving a dose of DPT-containing vaccine among children 12-35 months: an analysis of 81 low- and middle-income countries (vol 14, pg 1490, 2024). J Epidemiol Glob Health. 2025;15(1).
- Zhao S, Liu S, Gao J, Ma N, Chen S, Chandan JS, Kim R, Karoli P, Niyi JL, et al. Prevalence of co-occurring forms of intimate partner violence against women aged 15–49 and the role of education-related inequalities: analysis of Demographic and Health Surveys. EClinicalMedicine. 2025;82.
- Eom YJ, Chi H, Bhatia A, Lee HY, Subramanian SV, Kim R. Individual- and community-level women's empowerment and complete use of maternal healthcare services: a multilevel analysis of 34 sub-Saharan African countries. Soc Sci Med. 2025;370:117816.
- Narayanan M, Karlsson O, Kumar A, Pullum TW, Kim R, Subramanian SV. Prevalence of severe and moderate anthropometric failure among children in India, 1993–2021. Matern Child Nutr. 2025;21(2):e13751.
- Oh H, Jo G, Kim OY, Lim H, Song SJ, Choi JH, Bae JH, Jin ES, Kim R, et al. Fact sheet: nationwide trends in dietary intakes among Korean adults, 2013–2022. Korean J Intern Med. 2025;40(3):427.
- Jeong J, Chi H, Bliznashka L, Pitchik HO, Kim R. Co-occurrence of stunting and off-track early child development in low- and middle-income countries. JAMA Netw Open. 2025;8(3):e2462263.
- Chen S, Ma N, Kong Y, Chen Z, Niyi JL, Karoli P, Msuya HM, Zemene MA, et al. Prevalence, disparities, and trends in intimate partner violence against women living in urban slums in 34 low-income and middle-income countries: a multi-country cross-sectional analysis. EClinicalMedicine. 2025;81.
- Sadhu R, Kim R, Subramanian SV. Severe food insecurity among middle-aged and older adults in India: insights from the Longitudinal Aging Study in India. Glob Food Secur. 2025;44:100822.
- Jung S, Lee HY, Choe S, Oh H, Subramanian SV, Kim R. Maternal media exposure and child anthropometric failures across 40 low- and middle-income countries. SSM Popul Health. 2025;29:101746.
- Ko S, Oh H, Subramanian SV, Kim R. Life course social mobility and cognitive function among middle-aged and older adults in India: exploring heterogeneity by gender. Soc Sci Med. 2025;366:117640.
- Novak D, Čule M, Kim J, Kim R, Subramanian SV. Individual versus group exercise effect on youth physical activity levels: a randomised controlled trial. BMJ Open Sport Exerc Med. 2025;11(1).
- Novak D, Petrušič T, Čule M, Milinović I, Kim J, Kim R, Subramanian SV. Building social capital in university students: a physical education intervention program. J Phys Act Health. 2025;22(2):270-280.
- Jain A, Kim R, Subramanian SV. Analyzing changes in types of household sanitation among 543 Parliamentary Constituencies between 2016 and 2021. PLOS Water. 2025;4(8):e0000409.
- Shah A, Kim R, Subramanian SV. Spatiotemporal changes in district-level carbon emissions in India, 2019–2024. Glob Sustain. 2025;8:e21.
- Johri M, Rajpal S, Kim R, Subramanian SV. Small-area variation in child under-vaccination in India: a multilevel analysis of cross-sectional data from 36 states and Union Territories, 707 districts, and 22,349 small areas. Lancet Reg Health Southeast Asia. 2025;32.
- Karlsson O, Rajpal S, Johri M, Kim R, Subramanian SV. Prevalence and trends of not receiving a dose of DPT-containing vaccine among children 12–35 months: an analysis of 81 low- and middle-income countries. J Epidemiol Glob Health. 2024;14(4):1490-1503.
- Karlsson O, Benski C, Kapoor M, Kim R, Subramanian SV. Association between neonatal mortality and births not weighed among 400 thousand institutional deliveries in 32 low- and middle-income countries. J Public Health (Oxf). 2024;46(4):e614-e622.

- Ambade M, Kim R, Subramanian SV. Experience of health care utilization for inpatient and outpatient services among older adults in India. Public Health Pract. 2024;8:100541.
- Choo S, Kim R, Lee H, Eom YJ, Yi H, Kim R, Williams DR, Kim SS. Heightened vigilance and its associations with suicidal ideation and suicide attempt among 285 Korean transgender and nonbinary adults: effect modification by connectedness to community. Suicide Life Threat Behav. 2024;54(6):993-1005.
- Lippert AM, Corsi DJ, Kim R, Wedow R, Kim J, Taddess B, et al. Polygenic and socioeconomic contributions to nicotine use and cardiometabolic health in early mid-life. Nicotine Tob Res. 2024;26(12):1616-1625.
- Karlsson O, Kim R, Subramanian SV. International trends in zinc treatment for diarrhea. Pediatrics. 2024;154(5):e2024066701.
- Lee HY, Kumar A, Jain A, Kim R, Subramanian SV. Trends in the quality of antenatal care in India: patterns of change across 36 states and union territories, 1999–2021. J Glob Health. 2024;14:04188.
- Sung M, Jain A, Kumar A, Kim R, Kulkarni B, Subramanian SV. Patterns of change in the association between socioeconomic status and body mass index distribution in India, 1999–2021. J Glob Health. 2024;14:04171.
- Ambade M, Mishra R, Kim R, Subramanian SV. Association between 23 correlates anthropometric failure among children: analysis of 2016 and 2021 National Family Health Surveys in India. Coll Antropol. 2024;48(3):197-224.
- Puno A, Jeong J, Bhatia A, Kim R. Violence against children and later substance use in low- and middle-income countries. Child Abuse Negl. 2024;155:106981.
- Kim J, Eom YJ, Ko S, Subramanian SV, Kim R. Problems accessing health care and under-5 mortality: a pooled analysis of 50 low- and middle-income countries. J Public Health (Oxf). 2024;46(3):315-325.
- Jain A, Harrison C, Kumar A, Kim R, Subramanian SV. Examining geographic variation in the prevalence of household drainage types across India in 2019-2021. NPJ Clean Water. 2024;7(1):71.
- Jain A, Kim R, Swaminathan S, Subramanian SV. Socioeconomic inequality in child health outcomes in India: analyzing trends between 1993 and 2021. Int J Equity Health. 2024;23(1):149.
- Puno A, Kim J, Bhatia A, Jeong J, Kim R. Violence against children, self-harm, and suicidal behaviors: a pooled and country-specific analysis of eight low- and middle-income countries. J Adolesc Health. 2024;75(1):60-68.
- Eom YJ, Chi H, Jung S, Kim J, Jeong J, Subramanian SV, Kim R. Women's empowerment and child anthropometric failures across 28 sub-Saharan African countries: a cross-level interaction by Gender Inequality Index. SSM Popul Health. 2024;26:101651.
- Sung M, Kumar A, Mishra R, Kulkarni B, Kim R, Subramanian SV. Temporal change in prevalence of BMI categories in India: patterns across States and Union territories of India, 1999–2021. BMC Public Health. 2024;24(1):1322.
- Subramanian SV, Kumar A, Pullum TW, Ambade M, Rajpal S, Kim R. Early-neonatal, late-neonatal, postneonatal, and child mortality rates across India, 1993-2021. JAMA Netw Open. 2024;7(5):e2410046.
- Jung S, Chi H, Eom YJ, Subramanian SV, Kim R. Multilevel analysis of determinants in postnatal care utilisation among mother-newborn pairs in India, 2019–21. J Glob Health. 2024;14:04085.
- Chi H, Jung S, Subramanian SV, Kim R. Socioeconomic and geographic inequalities in antenatal and postnatal care components in India, 2016–2021. Sci Rep. 2024;14(1):10221.
- Choo S, Kim R, Lee H, Eom YJ, Yi H, Kim R, Kim SS. Associations between discrimination experiences and symptoms of depression and anxiety among transgender adults: a nationwide cohort study of 269 transgender adults in South Korea. Soc Psychiatry Psychiatr Epidemiol. 2024;59(5):859-869.
- Devaraj K, Gausman J, Mishra R, Kumar A, Kim R, Subramanian SV. Trends in prevalence of unmet need for family planning in India: patterns of change across 36 States and Union Territories, 1993–2021. Reprod Health. 2024;21(1):48.

- Kim R, Choo S, Lee H, Eom YJ, Yi H, Kim R, Kim SS. Does discrimination prevent transgender and gender diverse people from seeking healthcare?: a nationwide cohort study in South Korea. Int J Transgend Health. 2024;25(2):283-294.
- Sung M, Kim R, Subramanian SV. The gender distribution and association between sociodemographic factors and self-injury: analysis from the Discharge Injury Patient Survey, South Korea. 2024.
- Jain A, Sharma S, Kim R, Subramanian SV. Food deprivation among adults in India: an analysis of specific food-categories, 2016-2021 (vol 66, 102313, 2023). EClinicalMedicine. 2024;69.
- Subramanian SV, Patnaik A, Kim R. Call for action: presenting constituency-level data on population, health and socioeconomic wellbeing related to 2030 Sustainable Development Goals for India. Lancet Reg Health Southeast Asia. 2024;22.
- Eom YJ, Lee H, Choo S, Kim R, Yi H, Kim R, Kim SS. Situational avoidance and its association with mental health among transgender adults in South Korea: a nationwide cohort study. LGBT Health. 2024;11(2):122-130.
- Karlsson O, Kim R, Subramanian SV. Prevalence of children aged 6 to 23 months who did not consume animal milk, formula, or solid or semisolid food during the last 24 hours across low- and middle-income countries. JAMA Netw Open. 2024;7(2):e2355465.
- Chen Z, Sharma S, Chen S, Kim R, Subramanian SV, Li Z. Prevalence, trend, and inequality of prolonged exclusive breastfeeding among children aged 6–23 months old in India from 1992–2021: a cross-sectional study of nationally representative data. J Glob Health. 2024;14:04026.
- Gausman J, Kim R, Kumar A, Ravi S, Subramanian SV. Prevalence of girl and boy child marriage across states and Union Territories in India, 1993–2021: a repeated cross-sectional study. Lancet Glob Health. 2024;12(2):e271-e281.
- Ko S, Park S, Kim J, Subramanian SV, Kim R. Spousal multimorbidity and depressive symptoms among older Indian couples: do one’s own health status and sex matter? GeroScience. 2024;46(1):885-896.
- Chi H, Eom YJ, Jung S, Kim J, Jeong J, Kim R. Maternal decision-making power and care-seeking behaviors for acutely ill children: a multilevel analysis of 33 Sub-Saharan African countries. Am J Trop Med Hyg. 2024;110(2):370.
- Cho Y, Ryu S, Kim R, Shin MJ, Oh H. Ultra-processed food intake and risk of type 2 diabetes in Korean adults. J Nutr. 2024;154(1):243-251.
- Jain A, Sharma S, Kim R, Subramanian SV. Food deprivation among adults in India: an analysis of specific food categories, 2016–2021. EClinicalMedicine. 2023;66.
- Ko S, Oh H, Subramanian SV, Kim R. Small area geographic estimates of cardiovascular disease risk factors in India. JAMA Netw Open. 2023;6(10):e2337171.
- deSouza PN, Chaudhary E, Dey S, Ko S, Németh J, Guttikunda S, et al. An environmental justice analysis of air pollution in India. Sci Rep. 2023;13(1):16690.
- Ambade M, Kim R, Subramanian SV. Socio-economic distribution of modifiable risk factors for cardiovascular diseases: an analysis of the national longitudinal ageing study in India. Prev Med. 2023;175:107696.
- Ko S, Kim R, Subramanian SV. Patterns in child health outcomes before and after the COVID-19 outbreak in India. JAMA Netw Open. 2023;6(6):e2317055.
- Subramanian SV, Ambade M, Kumar A, Chi H, Joe W, Rajpal S, Kim R. Progress on Sustainable Development Goal indicators in 707 districts of India: a quantitative mid-line assessment using the National Family Health Surveys, 2016 and 2021. Lancet Reg Health Southeast Asia. 2023;13.
- Subramanian SV, Ambade M, Sharma S, Kumar A, Kim R. Prevalence of Zero-Food among infants and young children in India: patterns of change across the States and Union Territories of India, 1993–2021. EClinicalMedicine. 2023;58.
- Jain A, Pitchik HO, Harrison C, Kim R, Subramanian SV. The association between anthropometric failure and toilet types: a cross-sectional study from India. Am J Trop Med Hyg. 2023;108(4):811.
- Kapoor M, Ravi S, Kim R, Subramanian SV. Exercising in India: an exploratory analysis using The Time Use Survey, 2019. Coll Antropol. 2023;47(1):39-48.
- Rajpal S, Kumar A, Johri M, Kim R, Subramanian SV. Patterns in the prevalence of unvaccinated children across 36 states and union Territories in India, 1993-2021. JAMA Netw Open. 2023;6(2):e2254919.

- Jain A, Rajpal S, Rana MJ, Kim R, Subramanian SV. Small area variations in four measures of poverty among Indian households: econometric analysis of National Family Health Survey 2019–2021. Humanit Soc Sci Commun. 2023;10(1):18.

2. Please explain the key elements of your study design and the use of the available datasets that make your study an original scientific co ntribution

This study is a cross-sectional analysis using data from the 2022 Bangladesh Demographic and Health Survey (DHS), a nationally representative household survey. We analyzed a subsample of currently married women aged 15–49 years who completed the DHS long questionnaire containing mental health information. Women's empowerment was assessed using the globally validated Survey-based Women's emPowERment Index (SWPER), and depressive and anxiety symptoms were measured using Patient Health Questionnaire–9 (PHQ-9) and Generalized Anxiety Disorder–7 (GAD-7), respectively. The st udy provides an original contribution by examining the association between multiple domains of women's empowerment and depression and anxiety using nationally representative data in Bangladesh, a context where this relationship has been critically understudied.

3. Please list all publications that addressed similar research questions in the same dataset and indicate where you cited them in your paper

Publication	Where cited in the manuscript
Shawon MSR, Hossain FB, Ahmed R, Poly IJ, Hasan M, Rahman MR. Role of women empowerment on mental health problems and care-seeking behavior among married women in Nepal: secondary analysis of nationally representative data. Archives of Women's Mental Health. 2024;27(4):527-36.	Introduction (paragraphs 2–4); Discussion (paragraph 2)
Leight J, Pedehombga A, Ganaba R, Gelli A. Women's empowerment, maternal depression, and stress: Evidence from rural Burkina Faso. SSM-Mental Health. 2022;2:100160.	Introduction (paragraphs 2–4)
Scott S, Arrieta A, Kumar N, Menon P, Quisumbing A. Multidimensional predictors of common mental disorders among Indian mothers of 6-to 24-month-old children living in disadvantaged rural villages with women's self-help groups: A cross-sectional analysis. PLoS One. 2020;15(6):e0233418.	Introduction (paragraphs 2–4)
Fielding D, Lepine A. Women's Empowerment and Wellbeing: Evidence from Africa. The Journal of Development Studies. 2017;53(6):826-40.	Introduction (paragraphs 2–4)
Antabe R, Antabe G, Sano Y, Pienaah CKA. Women's household decision-making autonomy and mental health outcomes in Mozambique. Glob Ment Health. 2025;12:e40.	Introduction (paragraphs 2–4)
Yoshikawa K, Kamiya Y. Married couples' perceptions on women's autonomy and subjective well-being in Lao PDR. International Journal of Social Economics. 2019;46(6):792-804.	Introduction (paragraphs 2–4)
Haeri-Mehrizi A, Mohammadi S, Rafifar S, Sadighi J, Kermani RM, Rostami R, et al. Health literacy and mental health: a national cross-sectional inquiry. Scientific Reports. 2024;14(1):13639.	Introduction (paragraphs 2–4)
Yount KM, Dijkerman S, Zureick-Brown S, VanderEnde KE. Women's empowerment and generalized anxiety in Minya, Egypt. Social science & medicine. 2014;106:185-93.	Introduction (paragraphs 2–4)
Alam A, Prodhan ME, Akhter T, Sagar NI, Hasan MN. SWPER-Measured women's empowerment and mental health in Bangladesh: a cross-sectional study from 2022 demographic and health survey. BMC Women's Health. 2026;119.	Discussion (paragraph 2)

Esteban-Gonzalo S, Khan MH, Hasan MM, Díaz-Morales JF. Psychosocial predictors of women's mental health in Bangladesh: Evidence from BDHS-2022 data on fertility, empowerment, decision-making, child loss, and sex-affective behaviours. Public Health. 2026;254:106222.	Discussion (paragraph 2)

4. Please explain how you addressed multiple testing through an appropriately rigorous statistical threshold and indicate this in the methods section

Multiple statistical tests were conducted across different domains of women’s empowerment and mental health outcomes. Because the analyses were guided by *a priori* hypotheses derived from previous literature, formal adjustments for multiple comparisons were not applied (Rothman, 1990). Statistical significance was assessed using a conventional threshold of $p<0.05$, and results were interpreted with attention to the consistency and magnitude of associations rather than relying solely on p-values.

5. Please declare to what extent have AI chatbots been used in developing your paper and to which parts of the paper did they contribute

AI chatbots were used only for minor language editing and improving the clarity of English expression. No AI tools were used for study design, data analysis, or interpretation of results. All content was written and verified by the authors, who take full responsibility for the manuscript.

Note. Rothman, K. J. (1990). No adjustments are needed for multiple comparisons. *Epidemiology*, *1*(1), 43-46.

Table S2. STROBE Statement—Checklist of items that should be included in reports of cross-sectional studies

	Item No	Recommendation	Response
**Title and abstract**	1	(*a*) Indicate the study's design with a commonly used term in the title or the abstract	Done
		(*b*) Provide in the abstract an informative and balanced summary of what was done and what was found	Done
**Introduction**			
Background/rationale	2	Explain the scientific background and rationale for the investigation being reported	Done
Objectives	3	State specific objectives, including any prespecified hypotheses	Done
**Methods**			
Study design	4	Present key elements of study design early in the paper	Done
Setting	5	Describe the setting, locations, and relevant dates, including periods of recruitment, exposure, follow-up, and data collection	Done
Participants	6	(*a*) Give the eligibility criteria, and the sources and methods of selection of participants	Done
Variables	7	Clearly define all outcomes, exposures, predictors, potential confounders, and effect modifiers. Give diagnostic criteria, if applicable	Done
Data sources/ measurement	8*	For each variable of interest, give sources of data and details of methods of assessment (measurement). Describe comparability of assessment methods if there is more than one group	Done
Bias	9	Describe any efforts to address potential sources of bias	Done
Study size	10	Explain how the study size was arrived at	Done
Quantitative variables	11	Explain how quantitative variables were handled in the analyses. If applicable, describe which groupings were chosen and why	Done
Statistical methods	12	(*a*) Describe all statistical methods, including those used to control for confounding	Done
		(*b*) Describe any methods used to examine subgroups and interactions	Done

		(*c*) Explain how missing data were addressed	Done
		(*d*) If applicable, describe analytical methods taking account of sampling strategy	Done
		(*e*) Describe any sensitivity analyses	Done
**Results**			
Participants	13*	(a) Report numbers of individuals at each stage of study—eg numbers potentially eligible, examined for eligibility, confirmed eligible, included in the study, completing follow-up, and analysed	Done
		(b) Give reasons for non-participation at each stage	N/A
		(c) Consider use of a flow diagram	Done
Descriptive data	14*	(a) Give characteristics of study participants (eg demographic, clinical, social) and information on exposures and potential confounders	Done
		(b) Indicate number of participants with missing data for each variable of interest	Done
Outcome data	15*	Report numbers of outcome events or summary measures	Done
Main results	16	(*a*) Give unadjusted estimates and, if applicable, confounder-adjusted estimates and their precision (eg, 95% confidence interval). Make clear which confounders were adjusted for and why they were included	Done
		(*b*) Report category boundaries when continuous variables were categorized	Done
		(*c*) If relevant, consider translating estimates of relative risk into absolute risk for a meaningful time period	N/A
Other analyses	17	Report other analyses done—eg analyses of subgroups and interactions, and sensitivity analyses	Done
**Discussion**			
Key results	18	Summarise key results with reference to study objectives	Done
Limitations	19	Discuss limitations of the study, taking into account sources of potential bias or imprecision. Discuss both direction and magnitude of any potential bias	Done
Interpretation	20	Give a cautious overall interpretation of results considering objectives, limitations, multiplicity of analyses, results from similar studies, and other relevant evidence	Done

Generalisability	21	Discuss the generalisability (external validity) of the study results	Done
**Other information**			
Funding	22	Give the source of funding and the role of the funders for the present study and, if applicable, for the original study on which the present article is based	Done

* Give information separately for exposed and unexposed groups.

Reference: von Elm E, Altman DG, Egger M, et al. The Strengthening the Reporting of Observational Studies in Epidemiology (STROBE) statement: guidelines for reporting observational studies. *Lancet*. 2007;370(9596):1453–1457.

Note: An Explanation and Elaboration article discusses each checklist item and gives methodological background and published examples of transparent reporting. The STROBE checklist is best used in conjunction with this article (freely available on the Web sites of PLoS Medicine at http://www.plosmedicine.org/, Annals of Internal Medicine at http://www.annals.org/, and Epidemiology at http://www.epidem.com/). Information on the STROBE Initiative is available at www.strobe-statement.org.

Table S3. Coding structure for each of the 14 items used for construction of SWPER index

Domain	Variable	Coding
**Attitude to violence**	**Beating justified if:**	Yes = -1;<br>Don't Know = 0;<br>No = 1
	1. Wife goes out without telling husband	
	2. Wife neglects the children	
	3. Wife argues with husband	
	4. Wife refuses to have sex with husband	
	5. Wife burns the food	
**Social independence**	6. Frequency of reading newspaper or magazine	Not at all = 0;<br>Less than once a week = 1;<br>At least once a week = 2
	7. Woman's education in completed years of schooling	Years
	8. Education difference: woman's minus husband's years of schooling	Years
	9. Age difference: woman's minus husband's age	Years
	10. Age of woman at first cohabitation	Years
	11. Age of woman first birth	Years
**Decision-making**	**Who usually decides on:**	Husband/other alone = -1;<br>Joint or respondent alone = 1
	12. Women's health care	
	13. Large household purchases	
	14. Visits to family or relatives	

Note. Retrieved from Ewerling F, Raj A, Victora CG, Hellwig F, Coll CV, Barros AJ. SWPER Global: A survey-based women's empowerment index expanded from Africa to all low-and middle-income countries. Journal of global health 2020; 10(2).

Table S4. Multivariable associations between women's empowerment scores and mental health outcomes among married women in Bangladesh (N=18900)

	Depressive symptoms[a]		Anxiety symptoms[b]	
	β^c	95% CI	β^c	95% CI
**Attitude to violence**	0.92*	(0.85 - 0.98)	0.90**	(0.84 - 0.96)
**Social independence**	0.88**	(0.80-0.97)	0.85**	(0.75 – 0.95)
**Decision-making**	1.05	(0.95– 1.16)	1.10	(0.98 – 1.27)

Note. β=Beta coefficients; CI=Confidence Intervals.

*p<0.05; ** p<0.01; *** p<0.001.

a. Depressive symptoms were defined as having moderate to severe depression, indicated by a PHQ-9 score of 10 or greater.

b. Anxiety symptoms were defined as having moderate to severe anxiety, indicated by a GAD-7 score of 10 or greater.

c. Separate models were specified for each SWPER domain for each mental health outcome, accounting for the complex multistage sampling design of the DHS. Covariates included women's occupation (not working/housewife, agricultural, professional/technical/skilled, and services/others), husband's type of occupation (same categories with women's occupation), number of child aged <5 years (0, 1, 2, and 3+), number of household members (1-25), sex of household head (male and female), place of residence (urban and rural), household wealth index (poorest, poorer, middle, richer, and richest), religion (Islam, Hindu, Buddhist, and others), and Division (Barishal, Chattogram, Dhaka, Khulna, Mymensingh, Rajshahi, Rangpur, and Sylhet). Models for the attitude to violence and decision-making domains were additionally adjusted for women's age (15-19, 20-29, 30-39, and 40-49 years) and education (no education, primary, secondary, and higher).
